# MACPET: model-based analysis for ChIA-PET

**DOI:** 10.1093/biostatistics/kxy084

**Published:** 2019-01-30

**Authors:** Ioannis Vardaxis, Finn Drabløs, Morten B Rye, Bo Henry Lindqvist

**Affiliations:** 1 Department of Mathematical Sciences, Norwegian University of Science and Technology, N-7491 Trondheim, Norway; 2 Department of Clinical and Molecular Medicine, Norwegian University of Science and Technology, N-7491 Trondheim, Norway; 3 Department of Clinical and Molecular Medicine, Norwegian University of Science and Technology, N-7491 Trondheim, Norway and Clinic of Surgery, St. Olavs Hospital, Trondheim University Hospital, N-7030 Trondheim, Norway

**Keywords:** ChIA-PET, MACPET, Model-based clustering, Paired-end tags, Peak-calling algorithm

## Abstract

We present model-based analysis for ChIA-PET (MACPET), which analyzes paired-end read sequences provided by ChIA-PET for finding binding sites of a protein of interest. MACPET uses information from both tags of each PET and searches for binding sites in a two-dimensional space, while taking into account different noise levels in different genomic regions. MACPET shows favorable results compared with MACS in terms of motif occurrence and spatial resolution. Furthermore, significant binding sites discovered by MACPET are involved in a higher number of significant three-dimensional interactions than those discovered by MACS. MACPET is freely available on Bioconductor. ChIA-PET; MACPET; Model-based clustering; Paired-end tags; Peak-calling algorithm.

## 1. Introduction

In recent years, a lot of interest has been placed on understanding the three-dimensional structure of chromosomes inside the cell nucleus (Woodcock and Dimitrov, 2001; Dekker *and others*, 2002; Woodcock, 2006; Wei *and others*, 2006; Fraser and Bickmore, 2007; Fullwood *and others*, 2009), as genomes are organized as three-dimensional rather than linear structures in the nucleus of the cell (Woodcock, 2006). These structures play an important role in chromosomal activities such as transcription and regulation of gene expression (Woodcock and Dimitrov, 2001;Woodcock, 2006).

The ChIA-PET method allows for analysis of the three-dimensional structure of DNA associated with a protein of interest. It can be used for finding protein binding sites (PBSs) on the genome as well as potential chromatin interactions associated with these proteins. These interactions provide information on the three-dimensional genome structure (Fullwood *and others*, 2009).

Most ChIA-PET data have been generated from short sequencing reads, although there are also more recent protocols using long-reads and transposase-based library preparation. In the introduction we will, however, focus on the short-read protocol. ChIA-PET data contain short DNA sequences $\sim 20$ base pairs (bp), which are called tags. Each tag is ligated to a half-linker sequence, either A or B (often TAAG for linker A and ATGT for linker B). Each of these half-linkers contains the site for the restriction enzyme used to cut the sequences for releasing the tags, for example, GTTGGA for the Mmel restriction enzyme which cuts 20 bp from its restriction site to reveal the 20 bp long tag sequence. Pairs of tag-half-linker products are connected to each other by proximity ligation to form tag-linker-tag products named paired-end-tags (PETs). The final linker sequence reveals different combinations of the two half-linkers A and B (Li *and others*, 2010).

The combination of the half-linkers classifies the PETs into three categories. *Ambiguous* PETs are those for which any of their half-linkers is missing. *Chimeric* PETs are those with half-linkers A/B or B/A and are derived from random ligations between different ChIP complexes. Finally, *non-chimeric* PETs are those with half-linkers A/A or B/B. Only the non-chimeric PETs are considered for the ChIA-PET analysis (Li *and others*, 2010).

After classifying the PETs based on their half-linkers, the linker sequences are removed from the non-chimeric PETs (Li *and others*, 2010), and the tags of each PET are separately mapped on the genome (Chiu *and others*, 2006;Fullwood *and others*, 2009). The location to which the tags are mapped classifies the PETs into three categories (see Figure S1 in supplementary material available at *Biostatistics* online) (Li *and others*, 2010;Harbers and Kahl, 2012). *Self-ligated* PETs are products of self-circularization ligation of a single DNA fragment. They consist of tags which belong to the same chromosome and strand, and which have the same orientation and short genomic span between them. Furthermore, each PET has the same chance of being sequenced on both strands which will result in both tags being mapped either on Watson or on Crick strand. *Intra-chromosomal* PETs consist of tags which belong to the same chromosome, have long genomic distance between them, and may have been mapped on different strands or with different orientation. *Inter-chromosomal* PETs have the same characteristics as the intra-chromosomal ones, but their tags are mapped on different chromosomes. Intra- and inter-chromosomal PETs correspond to two DNA fragments from different genomic regions bound to the same protein of interest and ligated to each other during the ligation process (Li *and others*, 2010;Harbers and Kahl, 2012).

The process of creating ChIA-PET data is composed of many experimental steps, each of which may introduce some noise to the data (Fullwood *and others*, 2009; Li *and others*, 2010; Do *and others*, 2013). Noise PETs are usually defined as those that show a random positioning on the genome without creating significant peaks (Li *and others*, 2010). However, noise might not be evenly distributed across the genome, but might gather around certain regions (Do *and others*, 2013). This suggests different amounts of noise in different regions. Furthermore, noise can also be generated by PCR clonal amplification. These PETs might show significant overlap and be misclassified as PBSs by peak-calling algorithms. For reducing this kind of noise (Li *and others*, 2010) proposes merging all PETs for which both of their tags overlap $\pm 1$ bp with another PET’s tags.

Self-ligated PETs are used for identifying PBSs by finding significant peaks of overlapping PETs on the genome. The tags of those PETs would pile up creating two peaks, one upstream and one downstream from the true PBS location; the exact binding position exists somewhere between the two peaks (Li *and others*, 2010;Harbers and Kahl, 2012).

MACS is a widely used algorithm for finding PBSs on DNA for ChIP-Seq data using a non-parametric model (Zhang *and others*, 2008). However, MACS is also used for ChIA-PET data by using only the 5-end tag of each PET. Because PETs can be sequenced from either strand with the same probability, using only the 5-end tag of each PET would reveal an upstream and a downstream density of the Watson and Crick tags, respectively, with the PBS location positioned in the middle. MACS identifies and separates the two densities around each PBS by scanning the genome with a user-specified window. It then shifts the two densities towards each other to find the precise binding location. Finally, it merges candidate PBSs which overlap to create a single one (Zhang *and others*, 2008). However, if the window is too large the PBSs might be overestimated, and if it is too small the probability of false positives increases (Do *and others*, 2013). The choice of the window size might hence be a challenge for the user.

It would be reasonable to assume that using both tags of the self-ligated PETs provided by ChIA-PET would result in better identification of the PBSs. This is because if a PET belongs to a PBS, then both of its tags should be mapped around that PBS, irrespective of strand. Additionally, using a parametric statistical model which takes into account specific characteristics of the PBSs for identifying their location, might be more efficient than using a non-parametric model. To be more specific, it might be reasonable to expect that the distribution of the upstream peak of a PBS would be negatively-skewed towards the PBS and with longer left tail, since more upstream tags will be mapped on the left side of the PBS. Accordingly, the distribution of the downstream peak would be positively-skewed towards the PBS and with longer right tail, since more of the downstream tags will be mapped on the right side of the PBS. Predicting more accurate PBS locations should result in better discovery of significant interactions between these PBSs.

Intra- and inter-chromosomal PETs are used for finding interactions between PBSs, which are previously identified by the self-ligated PETs. Those interactions provide information about the three-dimensional structure of the genome and how it is folded in the nucleus of the cell (Li *and others*, 2010;Harbers and Kahl, 2012).

MANGO is a complete ChIA-PET pipeline, which uses MACS for finding significant PBSs and searches for significant interactions between these PBSs by taking the distance between them into account. Moreover, the user can choose which stage of the MANGO analysis to run, as well as provide PBSs found by algorithms other than MACS. ChIA-PET Tool (CPT) (Li *and others*, 2010) and ChIA-PET tool 2 (CPT2) (Li *and others*, 2017) are also two complete ChIA-PET pipelines which use MACS for identifying significant PBSs. CPT, however, does not consider the genomic distance between the pairs of the PBSs when discovering interactions, while CPT2 achieves this by using MICC (He *and others*, 2015) for calling for significant interactions. ChiaSig (Paulsen *and others*, 2014) is another algorithm for discovering significant interactions for ChIA-PET data, using the non-central hypergeometric distribution (Harkness, 1965). MANGO, however, has been shown to give more accurate results than the above mentioned algorithms in terms of interaction analysis (Phanstiel *and others*, 2015).

In this article, we present model-based analysis for ChIA-PET (MACPET), an efficient method for discovering PBSs using ChIA-PET data. MACPET uses both tags of each self-ligated PET and estimates the PBSs using two-dimensional parametric mixture models. Modeling the self-ligated PETs in two dimensions, one dimension for each tag, and representing them as dots in a two-dimensional space, ensures that in order for a self-ligated PET to belong to a PBS, both of its tags need to belong to it. MACPET identifies the upstream and downstream peaks of each PBS by taking into account potential skewness of the peaks. Since both tags of each self-ligated PET are used, MACPET does not use strand information of the tags. Furthermore, MACPET models non-overlapping genomic regions separately and evaluates noise locally, which results in better identification of noise PETs and excludes the need for user-specified values. Finally, MACPET also implements the preliminary stages of ChIA-PET analysis like linker identification, linker trimming, mapping to the reference genome, and PET classification. The output of MACPET can be directly used in the MANGO algorithm. MACPET is publicly available at Bioconductor. It is mainly implemented in C++ and thus is fast and supports all relevant platforms.

## 2. Methods

MACPET currently implements a four-stage analysis of ChIA-PET data. Each of these stages (0–3) is briefly discussed in the following Sections. Figure S2 in supplementary material available at *Biostatistics* online shows a complete MACPET pipeline.

### 2.1. Stage 0: Linker filtering

MACPET identifies the half-linkers and classifies the PETs as ambiguous, chimeric, or non-chimeric. It then removes the half-linker sequences from non-chimeric to reveal the two tags of each PET in the data. The trimmed non-chimeric PETs are used in the next stage of the analysis. This stage also removes PETs which include non-standard residues (e.g., the letter N).

### 2.2. Stage 1: Mapping to the genome

MACPET maps the tags of the non-chimeric PETs separately to the reference genome using the Bowtie algorithm (Langmead *and others*, 2009). First, the tags are mapped without allowing any mismatch and the uniquely mapped tags are kept. Then non-mapped tags are subject to a second run mapping with at most one mismatch, again keeping only the uniquely mapped tags. Note that this is the same process as the one proposed in Li *and others* (2010). PETs with both of their tags uniquely mapped with zero or one mismatch are used for constructing the paired-end BAM file which is used in subsequent stages. Finally, PETs with either tag overlapping any black listed regions of the corresponding genome are removed before continuing to the next stage of the analysis (ENCODE Project Consortium, 2012).

### 2.3. Stage 2: PET classification

MACPET classifies the PETs into self-ligated, intra- and inter- chromosomal. Inter-chromosomal PETs can be easily separated as the tags of each PET are mapped on different chromosomes. The length of a PET is defined as the distance between its tags. For separating the other two categories, MACPET plots the histogram of the log-lengths of the PETs (using a bandwidth of $100$ for each bin), spanning from the minimum to the maximum length of the PETs. It then applies the elbow method for finding a cut-off between the two populations. This is simply done by imagining a straight line connecting the highest density point on the histogram (peak) with the rightmost point on the histogram and then choosing the point on the histogram with the longest perpendicular distance from the straight line. This method is very simple, but it turns out to give stable results in terms of cut-offs between the different datasets. Other methods, such as mixture models, were also tested. We did not use them, however, because they are more random and give different results if run multiple times.

Thereafter, PETs for which both of their tags overlap with another PET’s tags ($\pm 1$ bp) are removed and only one of these PETs is kept for reducing noise by PCR amplification procedures.

### 2.4. Stage 3: Peak calling

At this stage MACPET uses only the self-ligated PETs for identifying candidate binding site locations. The genome is first segmented into non-overlapping regions, where each region has at least two overlapping self-ligated PETs. Each self-ligated PET in a region overlaps with at least one other self-ligated PET in the same region and no other self-ligated PET in any other region. In this way, MACPET can analyse each region separately and ensure that a binding event can only belong to one region. An example of a region can be seen in Figure S3 in supplementary material available at *Biostatistics* online.

#### 2.4.1. Distribution of tags in a protein binding site.

Self-ligated PETs which construct a PBS are products of the same type of protein which binds on approximately the same position across a set of identical genomes (Li *and others*, 2010). Therefore, it should be reasonable to expect that self-ligated PETs in a specific PBS would have approximately the same characteristics regarding the positions of their upstream and downstream tags. On the other hand, noise PETs should have characteristics which differ from those in a PBS.

Consider a PBS $g$ with $n_g$ self-ligated PETs and let $S_g=\left(s_1,...,s_{n_g}\right)$, where $s_i=\left(x_i,y_i\right)$ is the pair of upstream and downstream tags in self-ligated PET $i$, $i=\{1,...,n_g\}$. Although each tag of a self-ligated PET is mapped separately on the genome, with one tag not affecting the position of the other, we assume that $x_i<y_i$. That is, we sort the tags of each self-ligated PET in increasing order for better representing the left and right stream tags. This of course creates a dependency between $x_i$ and $y_i$.

Because the PBS should have two peaks, one on each of its sides, MACPET models the left and right peaks of the PBS as a two-dimensional skew generalized t-distribution (SGT). The one-dimensional SGT distribution is a five parameter distribution which models both skewness and long tails of the data (Arslan and Genç, 2009). It consists of three parameters $\mu \in \Re$, $\lambda \in (-1,1)$ and $\sigma \in \Re^{+}$ which represent the mode, skewness, and scale, respectively, and two parameters $p,q>0$ which are shape parameters. Here $\Re^{+}$ represents positive real numbers. It has been shown that for estimating the first three parameters, both of the shape parameters need to be known (Arslan and Genç, 2009). We choose $p=2$ which leads to a normal-type peak of the mode, and $q=1$ which leads to heavy and long tails (Arslan and Genç, 2009). The one-dimensional SGT density function is:

(2.1) \begin{equation*}\label{eq:PDFSGT}f_{SGT}(x;\theta)=\frac{\left(1+\frac{(x-\mu)^2}{\left(1+sgn(x-\mu)\lambda\right)^2\sigma^2}\right)^{-3/2}}{2\sigma}\end{equation*}

where $\theta=\left(\mu,\lambda,\sigma\right)$, $sgn(x)$ is the signum function which equals $1$ if $x>0$, $-1$ if $x<0$ and 0 if $x=0$.

MACPET assumes that the two-dimensional density of a self-ligated PET $i$, $i=1,...,n_g$, in PBS $g$ is $f_g(s_i;\theta_g)=\Lambda\left(\theta_g\right)f_{xg}(x_i;\theta_{xg})f_{yg}(y_i;\theta_{yg})$ if $x_i<y_i$ and 0 if $x_i\geq y_i$, where $f_{xg}(x_i;\theta_{xg})$ and $f_{yg}(y_i;\theta_{yg})$ have the SGT density given in equation (2.1). Moreover, $\theta_{g}=\left(\theta_{xg},\theta_{yg}\right)$ are the parameters of the PBS $g$, with $\theta_{xg}=\left(\mu_{xg},\lambda_{xg},\sigma_{xg}\right)$, and $\theta_{yg}=\left(\mu_{yg},\lambda_{yg},\sigma_{yg}\right)$ being the parameters of the upstream and downstream peaks of the PBS, respectively.

The term $\Lambda\left(\theta_g\right)=\left(\int_{-\infty}^{\infty}f_{yg}\left(y;\theta_{yg}\right)F_{xg}(y;\theta_{xg})dy\right)^{-1}$ ensures that the function $f_g(s_i;\theta_g)$ integrates to 1, and hence is a valid probability density. Here $F_{xg}$ is the cumulative distribution function of the SGT (see Section S1 in supplementary material available at *Biostatistics* online).

MACPET models the left stream, $x_g=\left(x_1,...,x_{n_g}\right)$, and right stream tags, $y_g=\left(y_1,...,y_{n_g}\right)$, as negative and positive skewed towards the PBS location, respectively. This is achieved by imposing a hierarchical structure where $\lambda_{xg}$ is restricted in the interval $(-1,0]$ with the density function $f_{\lambda_x}(\lambda_{xg})=\left(1+\lambda_{xg}\right)\left(-\lambda_{xg}\right)^{\alpha}$, while $\lambda_{yg}$ is restricted in the interval $[0,1)$ with the density function $f_{\lambda_y}(\lambda_{yg})=\lambda_{yg}^{\alpha}\left(1-\lambda_{yg}\right)$. The value $\alpha=39$ has been chosen in order to ensure that $\lambda_{xg}$ will tend towards $-1$, while $\lambda_{yg}$ will tend towards $+1$.

Additionally, for ensuring that the left peak will be on the left side of the PBS, and the right peak on the right side of the PBS, MACPET uses the reparametrization $\mu_{xg}=\mu_{yg}-k_g$ where $k_g\in \Re^{+}$.

The precise binding location is assumed to be between the two peak modes, that is $\left(\mu_{xg}+\mu_{yg}\right)/2$. Furthermore, a $95\%$ interval for the binding location is defined as $(Q_{Lxg}(0.05),Q_{Ryg}(0.95))$, where $Q_{Lxg}(0.05)$ is the $5\%$ quantile of the upstream peak and $Q_{Ryg}(0.95)$ is the $95\%$ quantile of the downstream peak (see Section S1 in supplementary material available at *Biostatistics* online).

#### 2.4.2. Modeling a region.

Consider a region with $N$ self-ligated PETs and $G$ PBSs. Let $S=\left(s_1,...,s_N\right)$ be the self-ligated PETs in the region, with $s_i$ defined as before and $i=1,...,N$. MACPET models the region as a mixture of $G$ clusters representing the PBSs and a noise cluster representing randomly distributed PETs in the region. That is, the density of $S$ in the region is $f(S;\theta,p)=p_0f_0(S)+\sum_{g=1}^Gp_gf_g(S;\theta_g)$, where $p=(p_0,...,p_G)$ and $p_g$ are the mixing probabilities of each cluster, which sum to $1$. Furthermore, $\theta=(\theta_1,...,\theta_G)$ and $g=1,...,G$ refer to the PBS clusters and $g=0$ refers to the noise cluster. The noise cluster is assumed to be uniformly distributed with density $f_0(x,y)=1/(2.5V)$ if $x<y$ and 0 if $x\geq y$, where $V$ is the two-dimensional volume of the region. Note that the constant $2.5$ increases the volume of the region and creates a slightly bigger area over the overlapping self-ligated PETs. By doing that, MACPET takes into account the noise level surrounding the region.

Taking into account the hierarchical structure for the $\lambda$ parameters mentioned earlier in the text, the observed log-likelihood of the region is Fraley and Raftery (2007):

(2.2) \begin{align*}\label{eq:LLop}\ell_{op}(\theta,p;S)&=\sum\limits_{i=1}^N\log\left\{p_0f_0(s_i)+\sum\limits_{g=1}^Gp_gf_{g}(s_i;\theta_g)\right\}+\sum\limits_{g=1}^G\log(f_{\lambda_{x}}(\lambda_{xg}))+\sum\limits_{g=1}^G\log(f_{\lambda_{y}}(\lambda_{yg}))\end{align*}

MACPET uses the Expectation/Conditional Maximization Either (ECME) algorithm for fitting the region model in equation (2.2) (Liu and Rubin, 1994). A detailed description of the estimation procedure can be found in Section S2 in supplementary material available at *Biostatistics* online.

#### 2.4.3. Inference.

For assessing the significance of each candidate binding event, MACPET considers the quantile functions of the estimated candidate PBSs. Consider a candidate PBS $g$ located at chromosome $C$ and let $(Q_{Lxg}(0.05),Q_{Rxg}(0.95))$, and $(Q_{Lyg}(0.05),Q_{Ryg}(0.95))$ be the $95\%$ confidence intervals for its upstream and downstream peaks, respectively (see Section S1 in supplementary material available at *Biostatistics* online). Furthermore, let $S_{xg}$ and $S_{yg}$ be the lengths of these intervals, and $N_{Cx}$ and $N_{Cy}$ be the total number of upstream and downstream tags on the chromosome $C$, respectively. Note that $N_{Cx}=N_{Cy}$.

he null hypothesis for the upstream tags ($H_{0xg}$) assumes that the number of tags in the upstream peak of $g$ is random, following a Poisson distribution with intensity $\lambda_{xg}=max(2,\lambda_{Cx},\lambda_{wx10},\lambda_{wx15})$. Here $\lambda_{Cx}=N_{Cx}S_{xg}/S_{C}$ is the expected number of upstream tags in the upstream peak, given the chromosome size $S_{C}$. Furthermore, $\lambda_{wx10}=N_{w10x}S_{xg}/(10S_{xg})$ and $\lambda_{wx15}=N_{w15x}S_{xg}/(15S_{xg})$ are the expected number of upstream tags in the upstream peak, by considering at a window of $10$ and $15$ times the size of the upstream peak, respectively. Furthermore, the constant $2$ ensures that at least two tags have to exist in the peak interval in order to be considered significant. The analogous hypothesis is assumed for the downstream tags ($H_{0yg}$) of $g$.

The null hypothesis for the candidate PBS $g$ ($H_{0g}$) assumes that $g$ is not a PBS but a random sample of overlapping self-ligated PETs. Intuitively, for $g$ not being a true PBS, both of its upstream and downstream peaks need to be randomly formed, that is both $H_{0xg}$ and $H_{0yg}$ are valid. Let $E_g$ be the event that $g$ is not a true PBS and $E_{xg}$ and $E_{yg}$ the events under $H_{0xg}$ and $H_{0yg}$, respectively. Then under $H_{0g}$, the upstream and downstream tags are assumed to be independent and thus $P(E_g)=P(E_{xg}\cap E_{yg})=P(E_{xg})P(E_{yg})$. Therefore, the *p*-value for $g$ can be defined as $p_g=p_{xg}p_{yg}$, where $p_{xg}$ and $p_{yg}$ are the *p*-values of the upstream and downstream peak, respectively. Finally, the *p*-values from all the PBSs are corrected using the Benjamini-Hochberg procedure (Benjamini and Hochberg, 1995).

Note that the quantile intervals are computed assuming $\Lambda\left(\theta_g\right)=1$. That is, the quantile intervals are found using the marginal distributions of $x_g$ and $y_g$ under the assumption of independence between them. We use this assumption because computing the marginal distributions of $x_g$ and $y_g$, in case of dependence between them, is computationally intensive. This should not be a big violation of the model, however, as the estimated $\Lambda\left(\theta_g\right)$ value for the majority of the PBS in each dataset is very close to $1$ (see Figure S4 in supplementary material available at *Biostatistics* online). There are a few values that deviate substantially from $1$, which could be the result of rounding errors, while computing the $\Lambda\left(\theta_g\right)$ integral. The reason that we still include the $\Lambda\left(\theta_g\right)$ term when finding candidate PBS in the previous step of the algorithm is that we observed an increase in the speed of the algorithm as well as a smoother convergence, while assuming that $\Lambda\left(\theta_g\right)=1$ led to almost identical results but with slower speed.

## 3. Results

We compare MACPET with MACS on six ChIA-PET datasets publicly available at NCBI (NCBI Resource Coordinators, 2016) (see Section S7 in supplementary material available at *Biostatistics* online). Table S1 in supplementary material available at *Biostatistics* online presents the datasets used and shows the results of the first three stages of MACPET analysis. Because MACS cannot filter, trim, map or classify the PETs, we used MACPET for these stages. The self-ligated PETs found by MACPET are then used in MACS for binding site analysis. In the main text, we only present results from the ESR1 (MCF-7), CTCF (MCF-7), and CTCF (K562) datasets. The results of the remaining datasets can be found in the supplementary material available at *Biostatistics* online. However, we will refer to them in the main text.

**Fig. 1.**
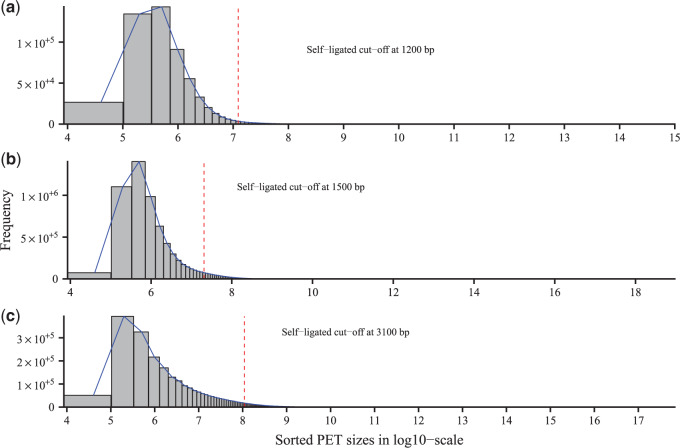
Self-intra cut-off. Self-ligated and intra-chromosomal cut-offs for the three datasets. (a) ESR1 (MCF-7), (b) CTCF (MCF-7), and (c) CTCF (K562). The *x*-axis are the lengths of the PETs in log10 scale, while the *y*-axis is the frequency. The dashed line represents the cut-off point, where the self-ligated PETs are on its left side and the intra-chromosomal on its right.

Figure 1 shows the self-ligated and intra-chromosomal separation cut-offs of the three datasets (for the other three datasets, see Figure S5 in supplementary material available at *Biostatistics* online). The self-ligated data were then used in both MACPET and MACS for finding significant PBSs in each dataset. For both MACPET and MACS we declared significant PBSs with false discovery rate (FDR) cut-off at $0.05$, mainly because this is the default cut-off for PBSs, which are used in the interaction analysis algorithm as we discuss later.

For the ESR1 (MCF-7), CTCF (MCF-7), and CTCF (K562) datasets we can investigate the association of the significant PBSs with the expected motifs (ESR1 and CTCT accordingly). Using $200$ bp windows centered at the summit of each PBS for the top $5000$ most significant PBSs from MACPET and MACS, we compared the quality and precision of these bindings in terms of motif occurrence (the percentage of PBSs associated with the expected motif) and spatial resolution (the distance of the PBS location to the expected motif). For doing so, we used the rGADEM algorithm (Droit *and others*, 2014) for *de novo* motif analysis, and then the MotIV algorithm (Mercier and Gottardo, 2014), for keeping only the most common motif on each dataset. rGADEM applies a stochastic algorithm for *de novo* motif discovery and it is therefore not guaranteed to give identical results on each run (Li, 2009). Therefore, we ran rGADEM five times for both MACPET and MACS (using MotIV after each run) and we took the mean among the runs as the final result. For motif occurrence we considered only those PBS which included the expected motif in a distance shorter than 50 bp. This is because these PBS show a stronger association to the expected motif as they are closer to it. Note that this is exactly what MACS does for comparing motif occurrences. For the spatial resolution, on the other hand, we used all the PBS which included the expected motif. If a PBS included the expected motif more than once, only the shortest distance was used. Finally, for both MACPET and MACS the most common motifs for each run were the expected motif for each dataset.

**Fig. 2.**
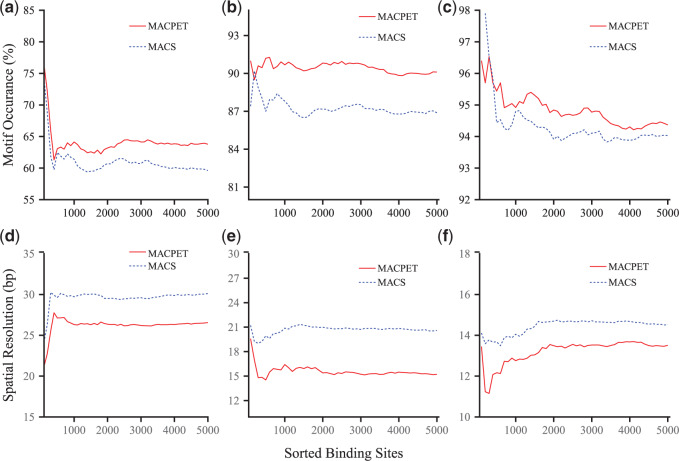
*De novo* motif discovery. Comparison of motif discovery and spatial resolution between MACPET and MACS. The *x*-axis for all plots is the top $5000$ PBSs, sorted by significance in descending order for each method respectively. (a–c) Motif occurrence (*y*-axis) for (a) ESR1 (MCF-7), (b) CTCF (MCF-7), (c) CTCF (K562). (d–f) Spatial resolution (*y*-axis) for (d) ESR1 (MCF-7), (e) CTCF (MCF-7), (f) CTCF (K562).

Figure 2(a–c) shows the motif occurrence for each dataset. MACPET results in a higher number of PBSs associated with the expected motif than those from MACS. Figure 2(d–f) shows the spatial resolution of the PBSs, where only PBSs with distance less than $50$ bp from the expected motif are taken into account. The locations of the MACPET PBSs are more precise as they are closer to the expected motif location than those of MACS.

For the results above, MACS was run on its default parameters. More specifically, the bandwidth (bw) was 300 bp and the size of the region, which is used around each peak for inference was 1000 bp (slocal). MACS has a lot of parameters which can be adjusted and might lead to different results. Therefore, we also tested MACS by altering some of its parameters which we thought might affect the results. More specifically, we let MACS denote the default parameters of MACS, MACS2 denote bw = 600 bp and slocal = 1000 bp, MACS3 denote bw = 1000 bp and llocal = 10 000 bp and MACS4 denote bw = 300 bp and llocal = 10 000 bp. We ran MACS for all these parameters and then discovered motifs using the same procedure as before. The results can be seen in Figure S6(a–f) in supplementary material available at *Biostatistics* online. As we can see, some parameter values perform better than the default parameters of MACS; however, none of them performs significantly better than MACPET.

**Fig. 3.**
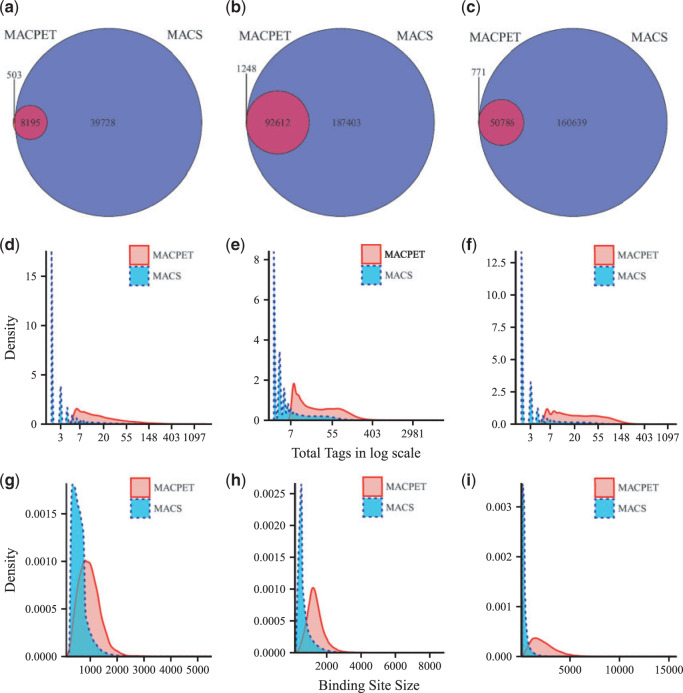
Comparison of significant binding sites. (a–c) Venn diagrams of the significant PBSs from MACPET and MACS for (a) ESR1 (MCF-7), (b) CTCF (MCF-7), (c) CTCF (K562). (d–f) densities for the total number of tags in each significant PBSs from MACPET and MACS for (d) ESR1 (MCF-7), (e) CTCF (MCF-7), (f) CTCF (K562). The *x*-axis is the total tags in log scale and the *y*-axis is the density of the total tags. (g–i) densities for the sizes of the significant PBSs from MACPET and MACS for (g) ESR1 (MCF-7), (h) CTCF (MCF-7), (i) CTCF (K562). The *x*-axis is the sizes of the significant PBSs and the *y*-axis is their density.

We also investigated the total common PBSs which MACPET and MACS found. Figure 3(a–c) shows the Venn diagrams for the significant PBSs, which are in common for MACPET and MACS, for the three datasets. For the rest of the datasets, see Figure S7(a–c) in supplementary material available at *Biostatistics* online. There are, in general, many PBSs which are common for the two algorithms. However, it is noticeable that MACS finds far more significant PBSs than MACPET. In Figure 3(d–f), on the other hand, one can see that MACPET finds stronger PBSs in terms of total tags than MACS for all six datasets. For the rest of the datasets, see Figure S7(d–f) in supplementary material available at *Biostatistics* online.

Additionally, comparing the interval sizes of the PBSs in Figure 3(g–i), we can see that MACPET seems to result in larger, but probably more realistic PBS intervals than MACS. For the rest of the datasets, see Figure S7(g–i) in supplementary material available at *Biostatistics* online.

Moreover, we used the fifth stage in MANGO algorithm for investigating the potential benefits of the PBSs from MACPET over those from MACS in terms of interaction analysis and three-dimensional DNA structure. We used the significant PBSs found by MACPET and MACS as inputs in MANGO (FDR $<0.05$, which is the default for peak-calling in MANGO), as well as the intra- and inter-chromosomal PETs classified by MACPET. Since MACS results in a higher number of significant PBS than MACPET (at the same FDR cut-off), which might affect the FDR of MANGO and, thus, the resulting interactions from MACS. Therefore, we also ran the fifth stage in MANGO algorithm using a lower FDR cut-off of $0.01$ for MACS, as well as using the top $N$ most significant PBS from MACS, where $N$ is the total significant PBS from MACPET at FDR level of $0.05$.

MANGO gives the option to extend the PBS intervals on both sides with a user specified window ($500$ bp being the default) (Phanstiel *and others*, 2015). Because MANGO merges the extended PBSs before running interaction analysis (Phanstiel *and others*, 2015), we ran MANGO on a sequence of extending windows $0,100,...,900,1000$. This allows us to investigate how different extending windows affect the merging of the PBSs and thus the interactions. The rest of MANGO parameters are kept at the default values for both MACPET and MACS.

**Fig. 4.**
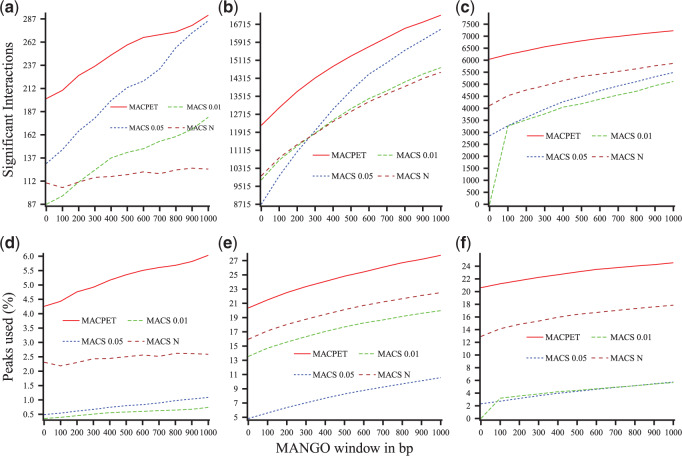
Comparison for MANGO interactions. Comparison of MANGO interaction results between significant PBSs from MACPET (peaks’ FDR $<0.05$) and MACS (peaks’ FDR $<0.05$, FDR $<0.01$ and top $N$ most significant peaks), for different PBSs extension windows. For all the plots, the *x*-axis is the number of bp. Each PBS interval was extended from either side before running MANGO. (a–c) Total significant interactions (*y*-axis) for (a) ESR1 (MCF-7), (b) CTCF (MCF-7), (c) CTCF (K562). (d–f) Proportion of significant PBSs involved in significant interactions (*y*-axis) for (d) ESR1 (MCF-7), (e) CTCF (MCF-7), (f) CTCF (K562).

Figure 4(a–c) shows the total number of significant interactions (FDR $<0.05$) for each extension window for MACPET and MACS, as well as for the lower FDR cut-off for MACS and the total number $N$ of significant MACS peaks. For the rest of the datasets, see Figure S8(a–c) in supplementary material available at *Biostatistics* online. MACPET gives higher total number of significant interactions than MACS for all six datasets, all windows and total peaks used. In Figure 4(d–f), we also compared the total PBSs involved in interactions for MACPET and MACS. For the rest of the datasets, see Figure S8(d–f) in supplementary material available at *Biostatistics* online. PBSs found by MACPET are more involved in interactions than those from MACS.

**Fig. 5.**
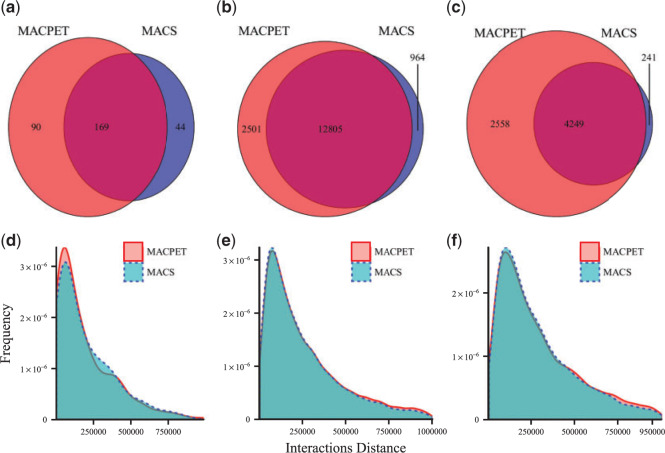
Comparison for MANGO interactions of $500$ bp window extension. (a–c) Venn diagrams from significant interactions for a $500$ bp extension window for the significant PBSs from MACPET and MACS for (a) ESR1 (MCF-7), (b) CTCF (MCF-7), (c) CTCF (K562). (d–f) Density plots for the distances of the significant intra-chromosomal interactions from MACPET and MACS for (d) ESR1 (MCF-7), (e) CTCF (MCF-7), (f) CTCF (K562). The *x*-axis is the sizes of the intra-chromosomal interactions and the *y*-axis is their density.

Finally, we considered only interactions for the $500$ bp extension window, for the peaks used at FDR cut-off of $0.05$ for both MACPET and MACS. Figure 5(a–c) shows the Venn diagrams for the common interactions between MACPET and MACS. For the rest of the datasets, see Figure S9(a–c) in supplementary material available at *Biostatistics* online. In general, there are many common interactions between MACPET and MACS. However, MACPET reveals many more interactions than MACS. Figure 5(d–f) shows the distance of the interactions, where MACPET seems to result in slightly longer interactions for all the datasets. For the rest of the datasets, see Figure S9(d–f) in supplementary material available at *Biostatistics* online.

## 4. Discussion

We compared MACPET with MACS, since the latter is one of the most used algorithms for discovering PBS. PICS (Zhang *and others*, 2011) is another known algorithm for binding-site analysis which also uses only the 5-end tag when used for ChIA-PET data. However, PICS needs control data for computing the FDR for the PBSs, and control data are unavailable for the datasets used in the analysis. Without an FDR estimate it was not possible to subset the most significant PBSs from PICS and thus, we could not compare PICS with MACPET.

We showed that MACPET discovers fewer significant PBSs than MACS. This is expected since MACPET models noise locally using mixture models, which results in weaker overlapping PETs being categorized as noise, while MACS categorizes them as PBSs. However, we showed that PBSs found by MACPET contain a higher number of tags than PBSs found by MACS, leading to stronger PBSs from MACPET. This is expected since MACPET forces both tags of each self-ligated PET to be part of a PBS.

Moreover, we showed that MACPET results in better identification or PBSs as well as more accurate positioning of PBSs than MACS. Although MACPET finds fewer significant PBSs, those PBSs are associated with the expected motif in higher frequency than those from MACS. This indicates once more that MACS has discovered a higher number of false PBSs with no motif association. Moreover, PBSs found by MACPET were closer to the exact motif position than those from MACS. Consequently, this confirms the assumption that using both tags of each PET in ChIA-PET data results in more accurate PBS locations. Note that even though MACPET results in broader PBS intervals than MACS, a 200 bp window centered at the PBS summit is used for both MACPET and MACS when searching for motifs. Therefore, the broadness of the PBS does not affect the motif discovery. Furthermore, no information about the total TAGs included in each PBS is used in the rGADEM algorithm since such information is not needed for motif discovery.

We also tested motif occurrence and spatial resolution for some different parameters of MACS, and we showed that none of these adjustments gives better performance than MACPET. The major ChIA-PET pipeline algorithms which use MACS on their peak-calling step, allow for adjustment of MACS’ parameters (see Li *and others*, 2010, 2017; Phanstiel *and others*, 2015). However, we believe that the user of such pipelines might find it challenging to alter those parameters and then re-run the interactions-calling step after each alteration. This problem can be avoided using MACPET because all of the MACPET parameters are learned and estimated from the data, eliminating the need for complex adjustments by the user.

Additionally, we showed that MACPET results in PBSs with longer and, overall, more flexible intervals. The skewness that MACPET implements when modeling PBSs seems to reflect the characteristics of the proteins being modeled. For example, PBSs from the datasets ESR1 (MCF-7), CTCF (MCF-7), and CTCF (K562), which are transcription factor proteins known to bind at specific locations, give smaller intervals than the dataset POL2 (K562), which is a polymerase protein known to bind in wide locations. MACS also captures the characteristics of the proteins, but not as much as MACPET does, because MACS’ model is not as flexible.

We also investigated how PBSs found by MACPET affect the three-dimensional genome interactions, compared with those from MACS. We used the significant PBSs found by MACPET and MACS in the interaction stage of the MANGO algorithm, using multiple significance thresholds for MACS. We showed that MACPET resulted in a higher number of significant interactions between its PBSs irrespective of the extending window, or the significance threshold used for MACS peaks. Furthermore, a higher proportion of PBSs found by MACPET are involved in interactions than those from MACS. This also indicates that the quality and precision of the PBSs found by MACPET are better than those from MACS.

Finally, we showed that PBSs found by MACPET are involved in slightly longer interactions than PBSs found by MACS. It is well known that PBSs, which are close to each other in genomic distance, tend to randomly interact more often than PBSs, which are separated by long genomic distance (Dekker *and others*, 2002). This also indicates that the PBSs found by MACPET are more accurate than those found by MACS.

## 5. Conclusions

The aim of this study was to create an algorithm-pipeline which would take advantage of all the available information provided by paired-end data such as ChIA-PET for discovering PBSs. The reason behind this was that identifying more accurate PBS locations should result in more robust identification of interactions. As intra- and inter-chromosomal PETs connect PBSs by being mapped near the PBSs’ binding locations, improperly identified PBSs might result in weak or even inaccurate interactions. We created MACPET, which runs a ChIA-PET data analysis including stages for linker trimming, mapping to the reference genome, PET classification, as well as a new statistical method for discovering PBSs using both tags of each PET. We showed that using all the available information from the paired-end data, combined with a more flexible model when discovering PBSs, is very important and leads to the discovery of a higher number of interactions between those PBSs. These interactions might reveal new insights of the three-dimensional DNA structure which might not have been found by using only the one tag of the paired-end data for finding PBSs. Finally, although the output from MACPET can be directly used in MANGO for interaction analysis, we are planning to implement a new interaction model in MACPET in the near future.

MACPET can handle both short-read, as well as long-read ChIA-PET data. Furthermore, it has the potential to be used in paired-end ChIP-seq as well as ATAC-Seq data (Buenrostro *and others*, 2013), by using both tags of such data for peak-calling instead of one. At this moment, the user can use Stage 3 for such data but the pre-processing of the data has to be done in advance because it is different than pre-processing of ChIA-PET data. In later updates of MACPET, we are planning to include pre-processing stages for such data as well.

## 6. Software

The MACPET algorithm is available on Bioconductor (https://bioconductor.org/packages/MACPET) under the public license GPL-3, as well as GitHub (https://github.com/IoannisVardaxis/MACPET). MACPET can be used on all platforms and supports parallelization. In this article, we used MACPET version $1.1.4$ in parallel on a 4 CPU Mac. Table S2 in supplementary material available at *Biostatistics* online shows the running time of each stage of MACPET for each data set. Finally, the R scripts used for running MACPET, MACS, and the comparisons between them are available at https://github.com/IoannisVardaxis/MACPET_comparing_code.

## Supplementary Material

kxy084_Supplementary_Materials
