# Supplementary material for: MACPET: model-based analysis for ChIA-PET
Source: Biostatistics. 2019 Jan 30;21(3):625–39. doi: 10.1093/biostatistics/kxy084 (PMC7308020; doi:10.1093/biostatistics/kxy084)
Supplement: kxy084_Supplementary_Materials [file kxy084_supplementary_materials.pdf]

Biostatistics (2018), **0**, 0, *pp.* 1–21
doi:10.1093/biostatistics/SupMaterial

Supplementary Materials for MACPET: Model-based Analysis for ChIA-PET

IOANNIS VARDAXIS*

Department of Mathematical Sciences, Norwegian University of Science and Technology, N-7491 Trondheim, Norway.

ioannis.vardaxis@ntnu.no

ivardaxis89@gmail.com

FINN DRABLØS

Department of Clinical and Molecular Medicine, Norwegian University of Science and Technology, N-7491 Trondheim, Norway.

MORTEN B. RYE

Department of Clinical and Molecular Medicine, Norwegian University of Science and Technology, N-7491 Trondheim, Norway.

Clinic of Surgery, St. Olavs Hospital, Trondheim University Hospital, N-7030 Trondheim, Norway.

BO HENRY LINDQVIST

Department of Mathematical Sciences, Norwegian University of Science and Technology, N-7491 Trondheim, Norway.

*To whom correspondence should be addressed.

© The Author 2018. Published by Oxford University Press. All rights reserved. For permissions, please e-mail: journals.permissions@oup.com

1. Quantile function for the SGT distribution

The cumulative distribution function of the SGT distribution with density given in equation (2.1) in the main text is easily seen to be:

$$F_{SGT}(x;\theta) = \begin{cases} \frac{1-\lambda}{2}\left(\frac{(x-\mu)}{\sqrt{(x-\mu)^2+(1-\lambda)^2\sigma^2}}+1\right), & \text{if } x \leqslant \mu \\ \frac{1-\lambda}{2}+\frac{1+\lambda}{2}\frac{(x-\mu)}{\sqrt{(x-\mu)^2+(1+\lambda)^2\sigma^2}}, & \text{otherwise} \end{cases}$$

Setting $F_{SGT}(x;\theta) = p$ for both cases and solving with respect to x gives the following quantile function:

$$Q(p) = \begin{cases} Q_L(p) = \mu - (1-\lambda)\sigma\left(\left(\frac{2p}{1-\lambda}-1\right)^{-2}-1\right)^{-1/2}, & \text{if } p \leqslant \frac{1-\lambda}{2} \\ Q_U(p) = \mu + 2\sigma\left(\left(p-\frac{1-\lambda}{2}\right)^{-2}-\frac{4}{(1+\lambda)^2}\right)^{-1/2}, & \text{otherwise} \end{cases}$$

where L refers to the lower and U to upper quantile.

2. Deriving the likelihood for the estimation

The SGT distribution can be represented as a mixture of a skew exponential power distribution (SEP) and a generalized gamma distribution (GG). Let $Y \sim SEP(1,2,\lambda)$, $Z \sim GG(1,1,1)$ be independent from each other. Then $X = \mu + \sigma Y Z^{-1/2}$ is SGT distributed with density given in equation (2.1) in the main text (Arslan and Genç (2009)). The joint density of X, Z is:

$$h_{SGT}(x,z;\theta) = \frac{\sqrt{z}}{\sqrt{\pi}\sigma}\exp\left\{-z\left(1+\frac{(x-\mu)^2}{(1+sgn(x-\mu)\lambda)^2\sigma^2}\right)\right\} \tag{E1}$$

where $\theta = (\mu, \lambda, \sigma)$

For the estimation purpose, MACPET uses this representation of the SGT distribution, since it makes the estimation procedure easier, as proposed by (Arslan and Genç (2009); Arslan (2010)). MACPET replaces $f_g(s_i;\theta_g)$ with $h_g(s_i, z_{gi};\theta_g)$, where $z_{gi} = (z_{xgi}, z_{ygi})$ are unobserved latent variables, $g = 1, ..., G$ and $i = 1, ..., N$. Moreover, $h_g(s_i, z_{gi};\theta_g) = \Lambda(\theta_g)\, h_{xg}(x_i, z_{xgi};\theta_{xg}) h_{yg}(y_i, z_{ygi};\theta_{yg})$, where $h_{xg}(x_i, z_{xgi};\theta_{xg})$ and $h_{yg}(y_i, z_{ygi};\theta_{yg})$ are joint densities as given in equation (E1).

Using the above representation and taking into account the hierarchical structure for the λ parameters given in the main text, the observed log-likelihood of the region becomes (Fraley and Raftery (2007)):

$$\ell_{op}(\theta, p; S, Z) = \sum_{i=1}^{N} \log \left\{ p_0 f_0(s_i) + \sum_{g=1}^{G} p_g h_g(s_i, z_{gi}; \theta_g) \right\} + \sum_{g=1}^{G} \log(f_{\lambda_x}(\lambda_{xg})) + \sum_{g=1}^{G} \log(f_{\lambda_y}(\lambda_{yg}))$$

where $Z = (z_0, ..., z_G)$ and $z_g = (z_{g1}, ..., z_{gN})$. Given unobserved latent variables $\gamma = (\gamma_1, ..., \gamma_G)$, the complete log-likelihood of the region is (Fraley and Raftery (2007)):

$$\ell_{cp}(\theta, p; S, Z, \gamma) = \sum_{i=1}^{N} \left(\gamma_{0i} \log \{p_0 f_0(s_i)\} + \sum_{g=1}^{G} \gamma_{gi} \log \{p_g h_g(s_i, z_{gi}; \theta_g)\} \right) + \sum_{g=1}^{G} \log(f_{\lambda_x}(\lambda_{xg})) + \sum_{g=1}^{G} \log(f_{\lambda_y}(\lambda_{yg})) \quad \text{(E2)}$$

with $\gamma_g = (\gamma_{g1}, ..., \gamma_{gN})$, where $\gamma_{gi} = 1$ if PET i belongs to cluster g and 0 otherwise.

To overcome the problem of the latent variables γ and Z, we consider the conditional expectation of equation (E2) given the observed data S. Keeping only terms which interact with the parameters, we get:

$$\begin{aligned} E(\ell_{cp}(\theta, p; S, Z, \gamma)|S) = &\sum_{g=0}^{G} n_g \log(p_g) + \sum_{g=1}^{G} n_g \log\left(\Lambda\left(\theta_g\right)\right) \\ &+ \sum_{i=1}^{N}\sum_{g=1}^{G} \hat{\gamma}_{gi} \left\{ \frac{\log(u_{xgi}) + \log(u_{ygi})}{2} - \log(\sigma_{xg}) - \log(\sigma_{yg}) \right. \\ &- u_{xgi} \left(1 + \frac{(x_i - \mu_{yg} + k_g)^2}{(1 + sgn(x_i - \mu_{yg} + k_g)\lambda_{xg})^2 \sigma_{xg}^2} \right) \\ &\left. -u_{ygi} \left(1 + \frac{(y_i - \mu_{yg})^2}{(1 + sgn(y_i - \mu_{yg})\lambda_{yg})^2 \sigma_{yg}^2} \right) \right\} \\ &+ \sum_{g=1}^{G} \{\log(1 + \lambda_{xg}) + 39\log(-\lambda_{xg}) + 39\log(\lambda_{yg}) + \log(1 - \lambda_{yg})\} \end{aligned} \quad \text{(E3)}$$

where $N = \sum_{g=0}^{G} n_g$, $n_g = \sum_{i=1}^{N} \hat{\gamma}_{gi}$, $\hat{\gamma}_{gi} = p_g f_g(s_i; \theta_g)/(\sum_{g=0}^{G} p_g f_g(s_i; \theta_g))$ and $f_g(\cdot)$ are the densities described in the main text (Fraley and Raftery (2007)). Furthermore, $u_{xgi} = E(z_{xgi}|x_i; \theta_{xg})$

and $u_{ygi} = E(z_{ygi}|y_i;\theta_{yg})$ (see Section 3: *Proof of* $E(z|x)$). There is no closed form solution of the derivative of equation (E3) with respect to any of the parameters. Therefore, the Expectation/Conditional Maximization Either (ECME) algorithm is used for the estimation (Liu and Rubin (1994)). For the estimation procedure see Section 4: *Derivation of ECME* and Section 6: *Initialization of ECME and post-processing.*

3. PROOF OF $E(z|x)$

The conditional density of $Z|X$ is easily seen to be:

$$f_{z|x}(z|x;\theta) = \frac{h_{SGT}(x,z;\theta)}{f_{SGT}(x;\theta)} = \frac{2\sqrt{z}}{\sqrt{\pi}} \exp\{-zA\} A^{3/2}$$

where $h_{SGT}(x,z;\theta)$ is the density in equation (E1), $f_{SGT}(x;\theta)$ is the density in equation (2.1) in the main text, and $A = 1 + (x-\mu)^2/\left((1+sgn(x-\mu)\lambda)^2\sigma^2\right)$. Then the expectation of $Z|X$ is:

$$E(z|x;\theta) = \int_0^\infty z f_{z|x}(z|x;\theta) = \frac{2}{\sqrt{\pi}} \int_0^\infty (zA)^{3/2} \exp\{-zA\}\, dz$$

Setting $k = zA$ gives:

$$E(z|x;\theta) = \frac{2}{A\sqrt{\pi}} \int_0^\infty k^{3/2} \exp\{-k\}\, dk = \frac{3}{A\sqrt{\pi}} \int_0^\infty k^{1/2} \exp\{-k\}\, dk$$

while setting $u = \sqrt{k}$ gives:

$$E(z|x;\theta) = \frac{6}{A\sqrt{\pi}} \int_0^\infty u^2 \exp\{-u^2\}\, du = \frac{3}{A\sqrt{\pi}} \int_0^\infty \exp\{-u^2\}\, du = \frac{3}{2A}$$

Therefore, $E(z|x;\theta) = \frac{3}{2}\left(1 + \frac{(x-\mu)^2}{(1+sgn(x-\mu)\lambda)^2\sigma^2}\right)^{-1}$.

4. DERIVATION OF ECME

Let t denote the t-th step of the ECME algorithm, and let $\theta_{xg}^{(t)} = \left(\mu_{xg}^{(t)}, \lambda_{xg}^{(t)}, \sigma_{xg}^{(t)}\right)$, $\theta_{yg}^{(t)} = \left(\mu_{yg}^{(t)}, \lambda_{yg}^{(t)}, \sigma_{yg}^{(t)}\right)$, where $\mu_{xg}^{(t)} = \mu_{yg}^{(t)} - k_g^{(t)}$, and $p_g^{(t)}$ be the parameters of this step. For obtaining

the maximum likelihood estimate (MLE) of each parameter at step $t+1$, we maximize equation (E3) with respect to each parameter, given the estimated parameters of the previous step t. The ECME algorithm proceeds with the following four steps in order find the MLE estimates:

Step 1 (Expectation) This step updates the following three quantities:

$$\Lambda\left(\theta_g^{(t)}\right) \text{ see Section 5: } \textit{Derivation of } \Lambda\left(\theta_g\right)$$

$$\hat{\gamma}_{gi}^{(t+1)} = \frac{p_g^{(t)} f_g(s_i;\theta_g^{(t)})}{\sum_{g=0}^{G} p_g^{(t)} f_g(s_i;\theta_g^{(t)})}$$

$$u_{xgi}^{(t+1)} = E(z_{xgi}|x_i;\mu_{xg}^{(t)},\lambda_{xg}^{(t)},\sigma_{xg}^{(t)}) = \frac{3}{2}\left(1+\frac{(x_i-\mu_{xg}^{(t)})^2}{(1+sgn(x_i-\mu_{xg}^{(t)})\lambda_{xg}^{(t)})^2\sigma_{xg}^{2(t)}}\right)^{-1}$$

$$u_{ygi}^{(t+1)} = E(z_{ygi}|y_i;\mu_{yg}^{(t)},\lambda_{yg}^{(t)},\sigma_{yg}^{(t)}) = \frac{3}{2}\left(1+\frac{(y_i-\mu_{yg}^{(t)})^2}{(1+sgn(y_i-\mu_{yg}^{(t)})\lambda_{yg}^{(t)})^2\sigma_{yg}^{2(t)}}\right)^{-1}$$

4.0.1 *Step 2 (Maximization)* This step updates $\hat{p}_g^{(t+1)}$, $\hat{\mu}_{yg}^{(t+1)}$, $\hat{k}_g^{(t+1)}$ and $\hat{\mu}_{xg}^{(t+1)}$. The score function with respect to $p_g^{(t+1)}$ and subject to $\sum_{g=0}^{G} p_g = 1$ is:

$$\frac{\partial E(\ell_{cp}(\theta,p;S,Z,\gamma)|S)}{\partial p_g^{(t+1)}} = \frac{n_g}{p_g^{(t+1)}} + C_p = 0$$

where C_p is the Lagrange multiplier term from the constraint $C_p\left(\sum_{g=0}^{G} p_g - 1\right)$. It is easy to see that $C_p = -N$ and that the MLE estimate becomes $\hat{p}_g^{(t+1)} = n_g/N$, where $n_g = \sum_{i=1}^{N}\hat{\gamma}_{gi}^{(t+1)}$.

The score function with respect to $\mu_{yg}^{(t+1)}$, conditioning on the previous estimates $\lambda_{xg}^{(t)}$, $\lambda_{yg}^{(t)}$, $\sigma_{xg}^{(t)}$ and $\sigma_{yg}^{(t)}$, is:

$$\frac{\partial E(\ell_{cp}(\theta,p;S,Z,\gamma)|S)}{\partial \mu_{yg}^{(t+1)}} = 2\sum_{i=1}^{N}\hat{\gamma}_{gi}^{(t+1)}\left(w_{xgi}^{(t+1)}(x_i-\mu_{yg}^{(t+1)}+k_g^{(t+1)}) + w_{ygi}^{(t+1)}(y_i-\mu_{yg}^{(t+1)})\right) = 0$$

where $w_{xgi}^{(t+1)} = u_{xgi}^{(t+1)}\left((1+sgn(x_i-\mu_{xg}^{(t)})\lambda_{xg}^{(t)})\sigma_{xg}^{(t)}\right)^{-2}$ and $w_{ygi}^{(t+1)} = u_{ygi}^{(t+1)}\left((1+sgn(y_i-\mu_{yg}^{(t)})\lambda_{yg}^{(t)})\sigma_{yg}^{(t)}\right)^{-2}$. This leads to the following MLE for $\mu_{yg}^{(t+1)}$:

$$\hat{\mu}_{yg}^{(t+1)} = \frac{A_{myg}^{(t+1)} + B_{myg}^{(t+1)}k_g^{(t+1)}}{C_{myg}^{(t+1)}} \tag{E4}$$

where $A_{myg}^{(t+1)} = \sum_{i=1}^{N} \hat{\gamma}_{gi}^{(t+1)}(w_{xgi}^{(t+1)} x_i + w_{ygi}^{(t+1)} y_i)$, $B_{myg}^{(t+1)} = \sum_{i=1}^{N} \hat{\gamma}_{gi}^{(t+1)} w_{xgi}^{(t+1)}$ and $C_{myg}^{(t+1)} = \sum_{i=1}^{N} \hat{\gamma}_{gi}^{(t+1)}(w_{xgi}^{(t+1)} + w_{ygi}^{(t+1)})$.

Moreover, the score function with respect to $k_g^{(t+1)}$, subject to $k_g \geqslant 0$ and conditioning on the previous estimates $\lambda_{xg}^{(t)}$, $\lambda_{yg}^{(t)}$, $\sigma_{xg}^{(t)}$ and $\sigma_{yg}^{(t)}$, is:

$$\frac{\partial E(\ell_{cp}(\theta, p; S, Z, \gamma)|S)}{\partial k_g^{(t+1)}} = -\sum_{i=1}^{N} \hat{\gamma}_{gi}^{(t+1)} \left(\phi_{xgi}^{(t+1)}(x_i - \hat{\mu}_{yg}^{(t+1)} + k_g^{(t+1)})\right) + C_{kg} = 0$$

where $\phi_{xgi}^{(t+1)} = 2w_{xgi}^{(t+1)}$ and C_{kg} is the Lagrange multiplier for the constraint $C_{kg} k_g^{(t+1)}$. The MLE estimate of $k_g^{(t+1)}$ becomes:

$$\hat{k}_g^{(t+1)} = -\frac{\sum_{i=1}^{N} \hat{\gamma}_{gi}^{(t+1)} \phi_{xgi}^{(t+1)}(x_i - \hat{\mu}_{yg}^{(t+1)}) + C_{kg}}{\sum_{i=1}^{N} \hat{\gamma}_{gi}^{(t+1)} \phi_{xgi}^{(t+1)}} \tag{E5}$$

It can easily be seen that $C_{kg} = \sum_{i=1}^{N} \hat{\gamma}_{gi}^{(t+1)} \phi_{xgi}^{(t+1)}(x_i - \hat{\mu}_{yg}^{(t+1)})$, which leads to $\hat{k}_g^{(t+1)} = 0$ if the constraint is violated. If the constraint is not violated then, $C_{kg} = 0$, and the estimate in equation (E5) after replacing $\hat{\mu}_{yg}^{(t+1)}$ from equation (E4) becomes:

$$\hat{k}_g^{(t+1)} = \left(\frac{A_{myg}^{(t+1)}}{C_{myg}^{(t+1)}} - \frac{\sum_{i=1}^{N} \hat{\gamma}_{gi}^{(t+1)} \phi_{xgi}^{(t+1)} x_i}{\sum_{i=1}^{N} \hat{\gamma}_{gi}^{(t+1)} \phi_{xgi}^{(t+1)}} \right) \left(1 - \frac{B_{myg}^{(t+1)}}{C_{myg}^{(t+1)}} \right)^{-1} \tag{E6}$$

Therefore, on that step the ECM updates first $\hat{p}_g^{(t+1)}$, then $\hat{k}_g^{(t+1)}$ using equation (E6), or setting $\hat{k}_g^{(t+1)} = 0$ in case equation (E6) is negative. Then it updates $\hat{\mu}_{yg}^{(t+1)}$ using equation (E4) and finally $\hat{\mu}_{xg}^{(t+1)} = \hat{\mu}_{yg}^{(t+1)} - \hat{k}_g^{(t+1)}$.

4.0.2 *Step 3 (Expectation)* This step updates the following four quantities:

$$\eta_{xgi}^{(t+1)} = E(z_{xgi}|x_i; \hat{\mu}_{xg}^{(t+1)}, \lambda_{xg}^{(t)}, \sigma_{xg}^{(t)}) = \frac{3}{2} \left(1 + \frac{(x_i - \hat{\mu}_{xg}^{(t+1)})^2}{(1 + sgn(x_i - \hat{\mu}_{xg}^{(t+1)})\lambda_{xg}^{(t)})^2 \sigma_{xg}^{2(t)}} \right)^{-1}$$

$$\eta_{ygi}^{(t+1)} = E(z_{ygi}|y_i; \hat{\mu_{yg}}^{(t+1)}, \lambda_{yg}^{(t)}, \sigma_{yg}^{(t)}) = \frac{3}{2} \left(1 + \frac{(y_i - \hat{\mu}_{yg}^{(t+1)})^2}{(1 + sgn(y_i - \hat{\mu}_{yg}^{(t+1)})\lambda_{yg}^{(t)})^2 \sigma_{yg}^{2(t)}} \right)^{-1}$$

$$\zeta_{xgi}^{(t+1)} = \frac{2\eta_{xgi}^{(t+1)}(x_i - \hat{\mu}_{xg}^{(t+1)})^2}{\left(1 + sgn(x_i - \hat{\mu}_{xg}^{(t+1)})\lambda_{xg}^{(t)}\right)^2}, \quad \zeta_{ygi}^{(t+1)} = \frac{2\eta_{ygi}^{(t+1)}(y_i - \hat{\mu}_{yg}^{(t+1)})^2}{\left(1 + sgn(y_i - \hat{\mu}_{yg}^{(t+1)})\lambda_{yg}^{(t)}\right)^2}$$

4.0.3 *Step 4 (Maximization)* The score function with respect to $\sigma_{xg}^{(t+1)}$, keeping the rest of the parameters at their last update, is:

$$\frac{\partial E(\ell_{cp}(\theta, p; S, Z, \gamma)|S)}{\partial \sigma_{xg}^{(t+1)}} = \sum_{i=1}^{N} \hat{\gamma}_{gi}^{(t+1)} \left(-\frac{1}{\sigma_{xg}^{(t+1)}} + \frac{\zeta_{xgi}^{(t+1)}}{\sigma_{xg}^{3(t+1)}} \right) = 0$$

which gives the following MLE:

$$\hat{\sigma}_{xg}^{(t+1)} = \sqrt{\frac{\sum_{i=1}^{N} \hat{\gamma}_{gi}^{(t+1)} \zeta_{xgi}^{(t+1)}}{\sum_{i=1}^{N} \hat{\gamma}_{gi}^{(t+1)}}}$$

and accordingly for $\hat{\sigma}_{yg}^{(t+1)}$.

Furthermore, the score function with respect to $\lambda_{xg}^{(t+1)}$, conditioning on $\hat{\mu}_{xg}^{(t+1)}$ and $\sigma_{xg}^{(t)}$, is:

$$\frac{\partial E(\ell_{cp}(\theta, p; S, Z, \gamma)|S)}{\partial \lambda_{xg}^{(t+1)}} = \sum_{i=1}^{N} \hat{\gamma}_{gi}^{(t+1)} \left(\frac{2\eta_{xgi}^{(t+1)}(x_i - \hat{\mu}_{xg}^{(t+1)})^2 sgn(x_i - \hat{\mu}_{xg}^{(t+1)})}{\sigma_{xg}^{2(t)} \left(1 + sgn(x_i - \hat{\mu}_{xg}^{(t+1)})\lambda_{xg}^{(t+1)}\right)^3} \right)$$
$$+ \frac{1}{1 + \lambda_{xg}^{(t+1)}} + \frac{39}{\lambda_{xg}^{(t+1)}} = 0$$

Let $A_{\lambda xg}^{+} = \sum_{i \in N^+} \hat{\gamma}_{gi}^{(t+1)} \eta_{xgi}^{(t+1)} (x_i - \hat{\mu}_{xg}^{(t+1)})^2$ and $A_{\lambda xg}^{-} = \sum_{i \in N^-} \hat{\gamma}_{gi}^{(t+1)} \eta_{xgi}^{(t+1)} (x_i - \hat{\mu}_{xg}^{(t+1)})^2$. Here $i \in N^+$ and $i \in N^-$ denote the observations for which $sgn(x_i - \hat{\mu}_{xg}^{(t+1)}) = 1$ and -1, respectively. Note that observations for which $sgn(x_i - \hat{\mu}_{xg}^{(t+1)}) = 0$ do not contribute to the score function. Then, multiplying the score function for $\lambda_{xg}^{(t+1)}$ with $(1 + \lambda_{xg}^{(t+1)})^3 (1 - \lambda_{xg}^{(t+1)})^3 \lambda_{xg}^{(t+1)} \sigma_{xg}^{2(t)}/2$, we get the following updating function:

$$f_{\lambda xg}(\lambda_{xg}^{(t+1)}) = \lambda_{xg}^{(t+1)}(1 - \lambda_{xg}^{(t+1)})^3 A_{\lambda_{xg}}^{+} - \lambda_{xg}^{(t+1)}(1 + \lambda_{xg}^{(t+1)})^3 A_{\lambda_{xg}}^{-}$$
$$+ \frac{\sigma_{xg}^{2(t)}}{2}(1 + \lambda_{xg}^{(t+1)})^2 (1 - \lambda_{xg}^{(t+1)})^3 \left(\lambda_{xg}^{(t+1)} + 39(1 + \lambda_{xg}^{(t+1)}) \right) = 0 \quad \text{(E7)}$$

The root of equation (E7) gives the MLE for $\hat{\lambda}_{xg}^{(t+1)}$ and can be found using the Newton-Rapshon optimization algorithm (NROA) (Fletcher (1987)). Let $\lambda_{xg}^{(t+1,0)} = \lambda_{xg}^{(t+1)}$ be the initial value for the NROA, then update using $\lambda_{xg}^{(t+1,j+1)} = \lambda_{xg}^{(t+1,j)} - f_{\lambda xg}(\lambda_{xg}^{(t+1,j)})/f'_{\lambda xg}(\lambda_{xg}^{(t+1,j)})$ for $j = 0$ until convergence. Here $f'_{\lambda xg}(\cdot)$ is the derivative of $f_{\lambda xg}(\cdot)$. When NROA has converged at stage $j = J$, set $\hat{\lambda}_{xg}^{(t+1)} = \lambda_{xg}^{(t+1,J)}$.

Similarly, for the MLE of $\hat{\lambda}_{yg}^{(t+1)}$, the updating equation for NROA is:

$$\begin{aligned} f_{\lambda yg}(\lambda_{yg}^{(t+1)}) &= \lambda_{yg}^{(t+1)}(1-\lambda_{yg}^{(t+1)})^3 A^{+}_{\lambda_{yg}} - \lambda_{yg}^{(t+1)}(1+\lambda_{yg}^{(t+1)})^3 A^{-}_{\lambda_{yg}} \\ &+ \frac{\sigma_{yg}^{2(t)}}{2}(1-\lambda_{yg}^{(t+1)})^2(1+\lambda_{yg}^{(t+1)})^3\left(39(1-\lambda_{yg}^{(t+1)}) - \lambda_{yg}^{(t+1)}\right) = 0 \end{aligned}$$

where $A^{+}_{\lambda yg} = \sum_{i\in N^+} \hat{\gamma}_{gi}^{(t+1)} \eta_{ygi}^{(t+1)}(y_i - \hat{\mu}_{yg}^{(t+1)})^2$ and $A^{-}_{\lambda yg} = \sum_{i\in N^-} \hat{\gamma}_{gi}^{(t+1)} \eta_{ygi}^{(t+1)}(y_i - \hat{\mu}_{yg}^{(t+1)})^2$.

5. Derivation of $\Lambda\left(\theta_g\right)$

It can easily be seen that the integral in $\Lambda\left(\theta_g^{(t)}\right) = \left(\int_{-\infty}^{\infty} f_{yg}\left(y;\theta_{yg}^{(t)}\right) F_{xg}(y;\theta_{xg}^{(t)})dy\right)^{-1}$ can be broken into six parts. Here $f_{yg}(y;\theta_{yg}) = f_{SGT}(y;\theta_{yg})$ and the probability density function $F_x(x;\theta_x) = F_{SGT}\left(x;\theta_x\right)$ can be found in Section 1: *Quantile function for the SGT distribution.* The six parts of the integral are the following:

$$\frac{1-\lambda_{xg}^{(t)}}{4\sigma_{yg}^{(t)}} \int_{-\infty}^{\mu_{xg}^{(t)}} \left(1 + \frac{(y-\mu_{yg}^{(t)})^2}{(1-\lambda_{yg}^{(t)})^2\sigma_{yg}^{2(t)}}\right)^{-3/2} dy \tag{E8}$$

$$+ \frac{1-\lambda_{xg}^{(t)}}{4\sigma_{yg}^{(t)}} \int_{\mu_{xg}^{(t)}}^{\mu_{yg}^{(t)}} \left(1 + \frac{(y-\mu_{yg}^{(t)})^2}{(1-\lambda_{yg}^{(t)})^2\sigma_{yg}^{2(t)}}\right)^{-3/2} dy \tag{E9}$$

$$+ \frac{1-\lambda_{xg}^{(t)}}{4\sigma_{yg}^{(t)}} \int_{\mu_{xg}^{(t)}}^{\infty} \left(1 + \frac{(y-\mu_{yg}^{(t)})^2}{(1+\lambda_{yg}^{(t)})^2\sigma_{yg}^{2(t)}}\right)^{-3/2} dy \tag{E10}$$

$$+ \frac{1-\lambda_{xg}^{(t)}}{4\sigma_{yg}^{(t)}} \int_{-\infty}^{\mu_{xg}^{(t)}} \left(1 + \frac{(y-\mu_{yg}^{(t)})^2}{(1-\lambda_{yg}^{(t)})^2\sigma_{yg}^{2(t)}}\right)^{-3/2} \frac{(y-\mu_{xg}^{(t)})}{\sqrt{(y-\mu_{xg}^{(t)})^2 + (1-\lambda_{xg}^{(t)})^2\sigma_{xg}^{2(t)}}} dy \tag{E11}$$

$$+ \frac{1+\lambda_{xg}^{(t)}}{4\sigma_{yg}^{(t)}} \int_{\mu_{xg}^{(t)}}^{\mu_{yg}^{(t)}} \left(1 + \frac{(y-\mu_{yg}^{(t)})^2}{(1-\lambda_{yg}^{(t)})^2\sigma_{yg}^{2(t)}}\right)^{-3/2} \frac{(y-\mu_{xg}^{(t)})}{\sqrt{(y-\mu_{xg}^{(t)})^2 + (1+\lambda_{xg}^{(t)})^2\sigma_{xg}^{2(t)}}} dy \tag{E12}$$

$$+ \frac{1+\lambda_{xg}^{(t)}}{4\sigma_{yg}^{(t)}} \int_{\mu_{xg}^{(t)}}^{\infty} \left(1 + \frac{(y-\mu_{yg}^{(t)})^2}{(1+\lambda_{yg}^{(t)})^2\sigma_{yg}^{2(t)}}\right)^{-3/2} \frac{(y-\mu_{xg}^{(t)})}{\sqrt{(y-\mu_{xg}^{(t)})^2 + (1+\lambda_{xg}^{(t)})^2\sigma_{xg}^{2(t)}}} dy \tag{E13}$$

The first three integrals (E8-10) are easily seen to be, respectively:

$$\frac{(1-\lambda_{xg}^{(t)})(1-\lambda_{yg}^{(t)})}{4}\left(1+\frac{\mu_{xg}^{(t)}-\mu_{yg}^{(t)}}{\sqrt{(\mu_{xg}^{(t)}-\mu_{xg}^{(t)})^2+(1-\lambda_{yg}^{(t)})^2\sigma_{yg}^{2(t)}}}\right)$$

$$\frac{(1-\lambda_{xg}^{(t)})(1-\lambda_{yg}^{(t)})}{4}\frac{\mu_{yg}^{(t)}-\mu_{xg}^{(t)}}{\sqrt{(\mu_{xg}^{(t)}-\mu_{xg}^{(t)})^2+(1-\lambda_{yg}^{(t)})^2\sigma_{yg}^{2(t)}}}$$

$$\frac{(1-\lambda_{xg}^{(t)})(1+\lambda_{yg}^{(t)})}{4}$$

The other three integrals can be approximated using Simpson's rule (Atkinson (2004)).

6. Initialization of ECME and post-processing

MACPET makes an initial classification of PETs into noise PETs and PETs coming from a binding site. Because noise PETs are more sparse through the region, MACPET uses the nearest neighbor clutter removal procedure (Byers and Raftery (1998)), for finding which PETs are gathered together, using the Euclidean distance between each PET and its second nearest neighbor PET.

The tags of the PETs which are classified as potential binding site PETs, are used in Normal-kernel estimations with a bandwidth of 50 bp (Hastie *and others* (2009)). This is done separately on both stream tags x and y. Upstream and downstream peaks which are found by the kernel estimation are paired together such that the upstream peak will be on the left side of the downstream peak. Unpaired peaks are discarded from the model. MACPET uses the paired peaks for initializing the estimation algorithm, and it estimates the region model using the whole region data. Note that the total number of paired peaks is the total number of binding sites G in the region.

After the parameter estimation is finished, overlapping binding sites (based on the μ_{xg} and μ_{yg} estimates) are merged in one cluster and the estimation procedure is run again for updating the parameters. The process is repeated until no binding sites are overlapping.

7. Availability of Data

The datasets used during the current study are available in the NCBI repository (NCBI Resource Coordinators (2016)). More specifically:

ChIA-PET dataset for ESR1 TF from MCF-7 human cell line (GEO:GSM970212), available at (https://www.ncbi.nlm.nih.gov/geo/query/acc.cgi?acc=GSM970212).

ChIA-PET dataset for CTCF TF from MCF-7 human cell line (GEO:GSM970215), available at (https://www.ncbi.nlm.nih.gov/geo/query/acc.cgi?acc=GSM970215).

ChIA-PET dataset for CTCF TF from K562 human cell line (GEO:GSM970216), available at (https://www.ncbi.nlm.nih.gov/geo/query/acc.cgi?acc=GSM970216).

ChIA-PET dataset for histone H3K4me1 from K562 human cell line (GEO:GSM1436263), available at (https://www.ncbi.nlm.nih.gov/geo/query/acc.cgi?acc=GSM1436263).

ChIA-PET dataset for histone H3K27ac from K562 human cell line (GEO:GSM1436262), available at (https://www.ncbi.nlm.nih.gov/geo/query/acc.cgi?acc=GSM1436262).

ChIA-PET dataset for POL2 from K562 human cell line (GEO:GSM970213), available at (https://www.ncbi.nlm.nih.gov/geo/query/acc.cgi?acc=GSM970213).

All processed data are available at https://figshare.com/projects/MACPET_Model-based_Analysis_for_ChIA-PET/29473.

References

Arslan, Olcay. (2010). An alternative multivariate skew laplace distribution: properties and estimation. *Statistical Papers* **51**(4), 865–887.

Arslan, Olcay and Genç, Ali İ. (2009). The skew generalized t distribution as the scale mixture of a skew exponential power distribution and its applications in robust estimation. *Statistics* **43**(5), 481–498.

Atkinson, K.E. (2004). *An Introduction to Numerical Analysis.* 111 River Street, New Jersey, NJ 07030-5774, USA: John Wiley & Sons (Asia) Pte Limited.

Byers, Simon and Raftery, Adrian E. (1998). Nearest-Neighbor Clutter Removal for Estimating Features in Spatial Point Processes. *Journal of the American Statistical Association* **93**(442), pp. 577–584.

Fletcher, R. (1987). *Practical methods of optimization*, Volume 1, Wiley-Interscience publication. 111 River Street, New Jersey, NJ 07030-5774, USA: Wiley.

Fraley, Chris and Raftery, Adrian E. (2007). Bayesian Regularization for Normal Mixture Estimation and Model-Based Clustering. *J. Classif.* **24**(2), 155–181.

Hastie, T., Tibshirani, R. and Friedman, J. (2009). *The Elements of Statistical Learning: Data Mining, Inference, and Prediction, Second Edition*, Springer Series in Statistics. 233 Spring Street, New York, NY 10013, USA: Springer New York.

Liu, Chuanhai and Rubin, Donald B. (1994). The ECME Algorithm: A Simple Extension of EM and ECM with Faster Monotone Convergence. *Biometrika* **81**(4), 633–648.

NCBI Resource Coordinators. (2016). Database resources of the National Center for Biotechnology Information. *Nucleic Acids Research* **44**(Database issue), D7–D19.

Tables

Table S1: Description of the datasets.

Name	GEO	PETs	Ambiguous	Chimeric	NN	Usable	Final PETs	Inter-chrom.	Intra-chrom.	Self-ligated
ESR1 (MCF-7)	GSM970212	26170257	165093	2492122	620598	22892444	6575793	1765891	164974	534284
CTCF (MCF-7)	GSM970215	119959634	7105105	11018238	317231	101519060	53526399	20630265	2729210	5872607
CTCF (K562)	GSM970216	195436387	42363105	9477776	1893878	141701628	80927884	2824894	1531945	2337518
H3K4me1 (K562)	GSM1436263	162190720	23144511	30577815	465092	108003302	38161523	31963485	2483809	2867067
H3K27ac (K562)	GSM1436262	165109173	32418202	53756946	683759	78250266	25168926	19265500	1770107	3167743
POL2 (K562)	GSM970213	37889691	3920503	409894	453521	33105773	17473165	2919406	4068174	7387093

Name of the datasets (Name), GEO number of the datasets (GEO) and total number of initial PETs of the datasets (PETs). Total PETs classified as Ambiguous, Chimeric, Usable (non-chimeric), as well as the total PETs with non-standard residues (NN). Total valid paired and mapped PETs used (Final PETs), as well as their classification into inter-chromosomal, intra-chromosomal and self-ligated.

Table S2: Running time of MACPET.

Stage	ESR1 (MCF-7)	CTCF (MCF-7)	CTCF (K562)	H3K4me1 (K562)	H3K27ac (K562)	POL2 (K562)
0	9.6 min	47.7 min	1.4 hours	2.6 hours	2.3 hours	17.8 min
1	54.8 min	5.3 hours	7.3 hours	7.5 hours	3.9 hours	1.7 hours
2	1.15 min	6.5 min	2.5 min	9.3 min	4.7 min	7.2 min
3	2.4 min	16.1 min	6 min	19.7 min	14.2 min	57.8 min
Total	1.13 hours	6.4 hours	8.8 hours	10.5 hours	6.5 hours	3 hours

Running time for the datasets used in analysis for each of the following stages of MACPET: 0-Linker filtering, 1-Mapping to the genome, 2-PET classification and 3-Peak calling.

Figures

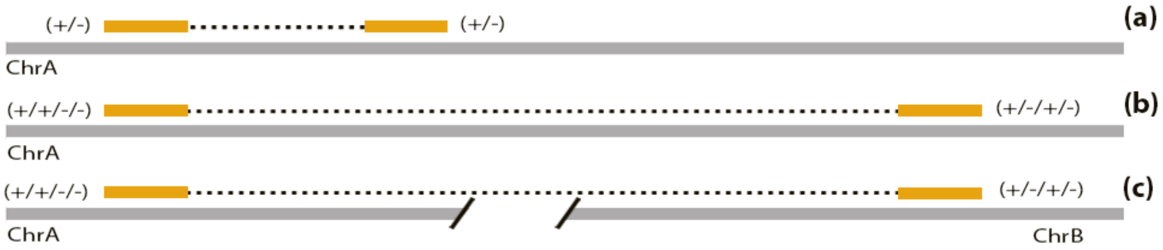

Fig. S1: Illustration of PET types. (a) Self-ligated PETs with both tags on the same chromosome and strand, and short genomic distance between them. (b) Intra-chromosomal PETs with tags on the same chromosome, with any strand combination and long genomic distance between them. (c) Inter-chromosomal PETs with tags on different chromosomes and with any strand combination.

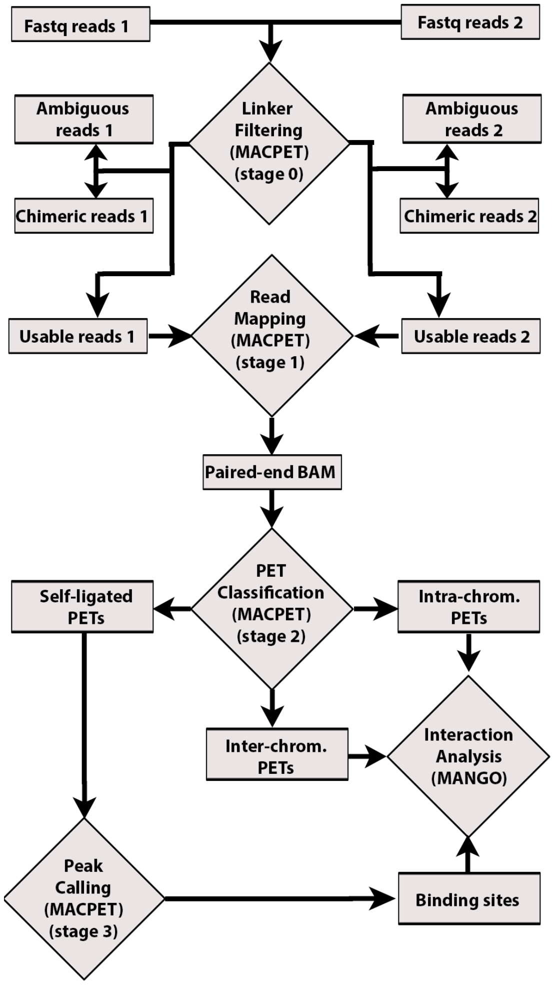

Fig. S2: MACPET pipeline. Stage 0: MACPET takes the forward (1) and reverse (2) fastq files as input, as well as the user-specified barcode sequences for the half-linkers. It then classifies the PETs as ambiguous, chimeric and usable (non-chimeric). The half-linkers of the usable PETs are trimmed to release the two tags of each PET. Stage 1: The tags of the usable PETs are mapped separately to the reference genome and a paired-end BAM file is created. Stage 2: PETs are classified as self-ligated, intra- and inter-chromosomal. Stage 3: Self-ligated PETs are used for discovering PBSs. Finally, the PBSs as well as the intra- and inter-chromosomal PETs can be used in MANGO for interaction analysis.

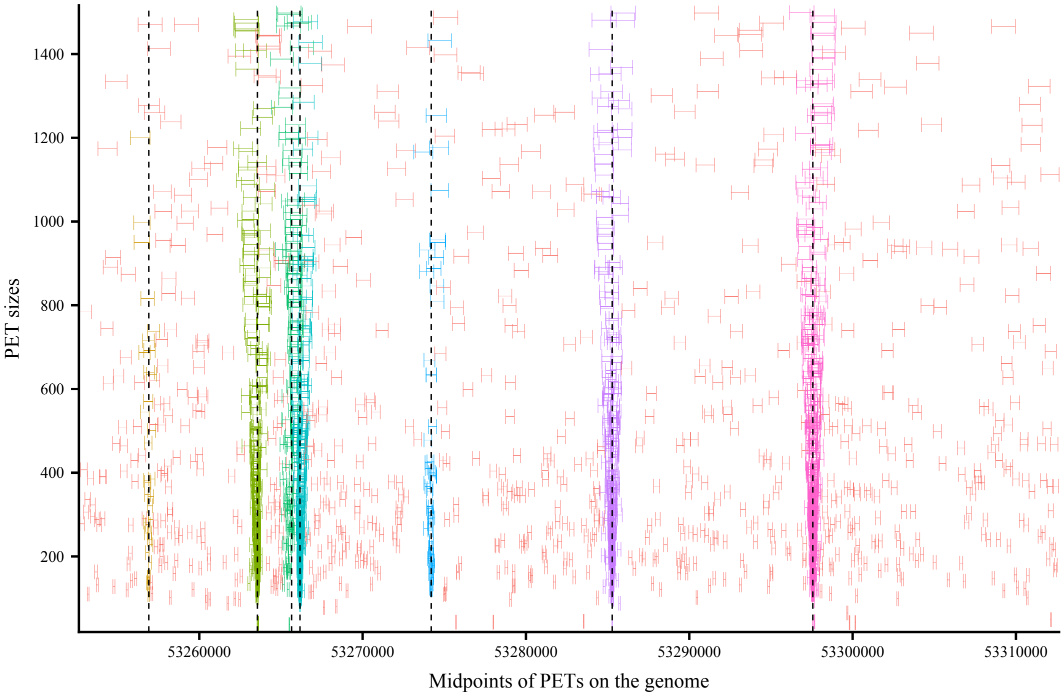

Fig. S3: Illustration of a region. Illustration of a region in two dimensions. The x-axis is the midpoints of the PETs and the y-axis is the length of the PETs. Each segment represents a PET from its upstream to its downstream tag. Red colored PETs are classified as noise PETs by MACPET. The rest colors represent binding sites, where each color represents a different binding site. The dashed lines represent the exact binding location found by MACPET for each binding site.

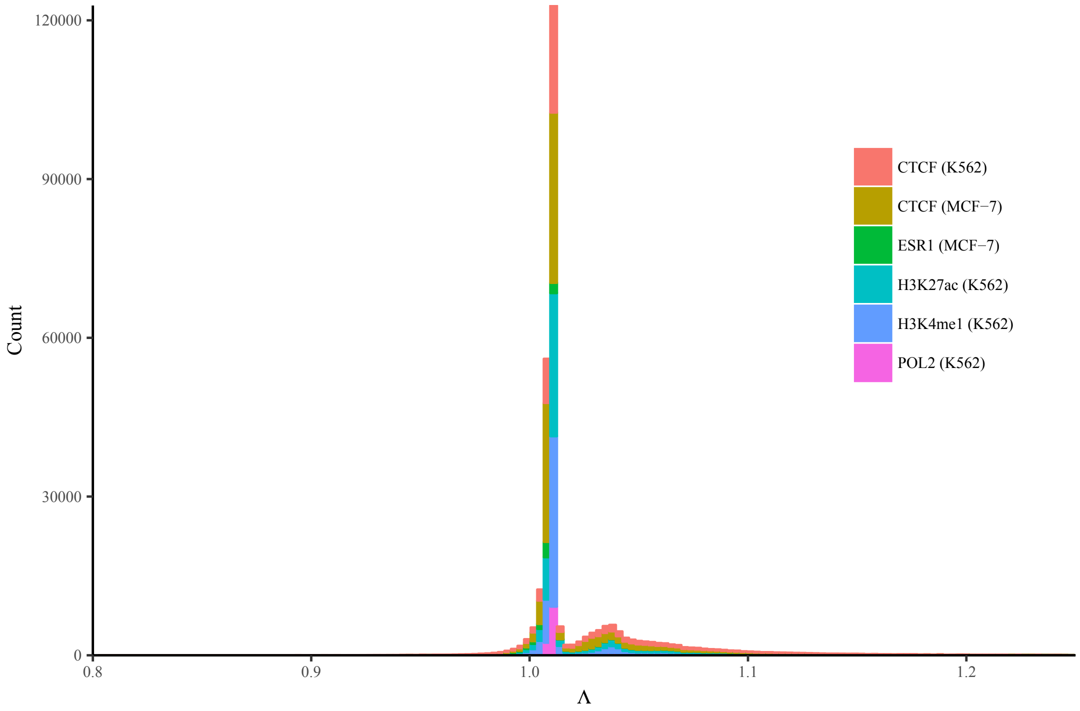

Fig. S4: Histogram for the $\Lambda(\theta_g)$ value. The x-axis gives the $\Lambda(\theta_g)$ value for each significant PBS in each dataset after running the ECME algorithm, while the y-axis shows their counts.

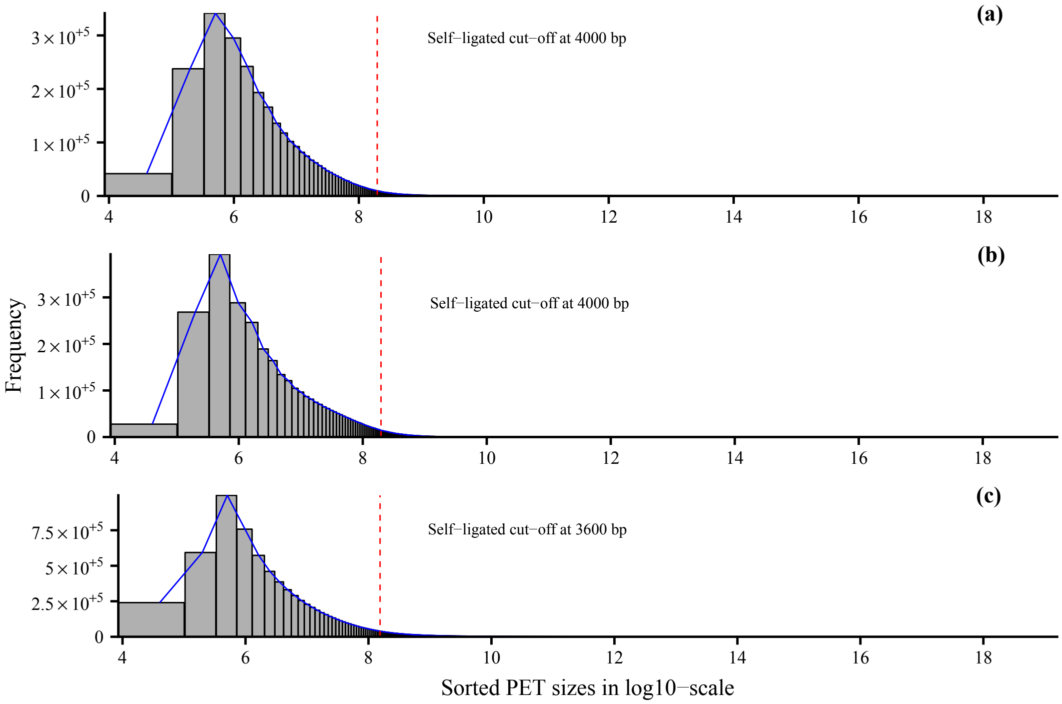

Fig. S5: Self-Intra cut-off. Self-ligated and Intra-chromosomal cut-offs for the three datasets (a) POL2 (K562), (b) H3K4me1 (K562), (c) H3K27ac (K562). The x-axis are the lengths of the PETs in log10 scale, while the y-axis is the frequency. The dashed line represents the cut-off point, where the self-ligated PETs are on the left side and the intra-chromosomal on the right.

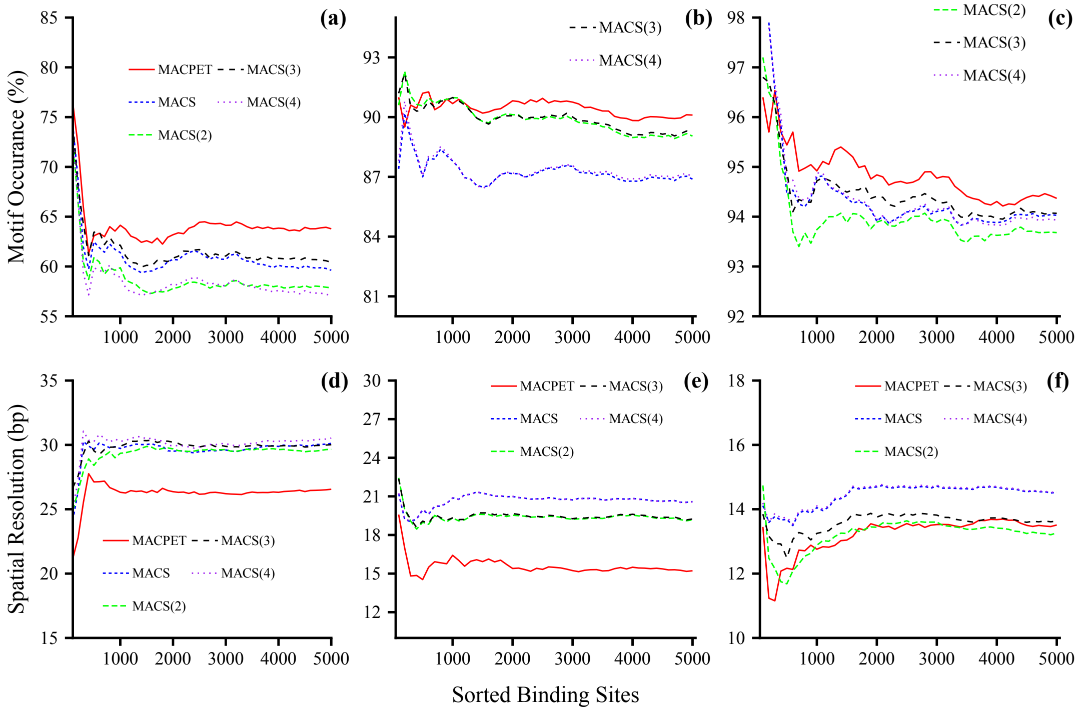

Fig. S6: De novo motif discovery for different MACS parameters. Comparison of motif discovery and spatial resolution between MACPET and different parameters for MACS. The x-axis for all plots is the top 5000 PBSs, sorted by significance in descending order for each method respectively. (a-c) Motif occurrence (y-axis) for (a) ESR1 (MCF-7), (b) CTCF (MCF-7), (c) CTCF (K562). (d-f) Spatial resolution (y-axis) for (d) ESR1 (MCF-7), (e) CTCF (MCF-7), (f) CTCF (K562).

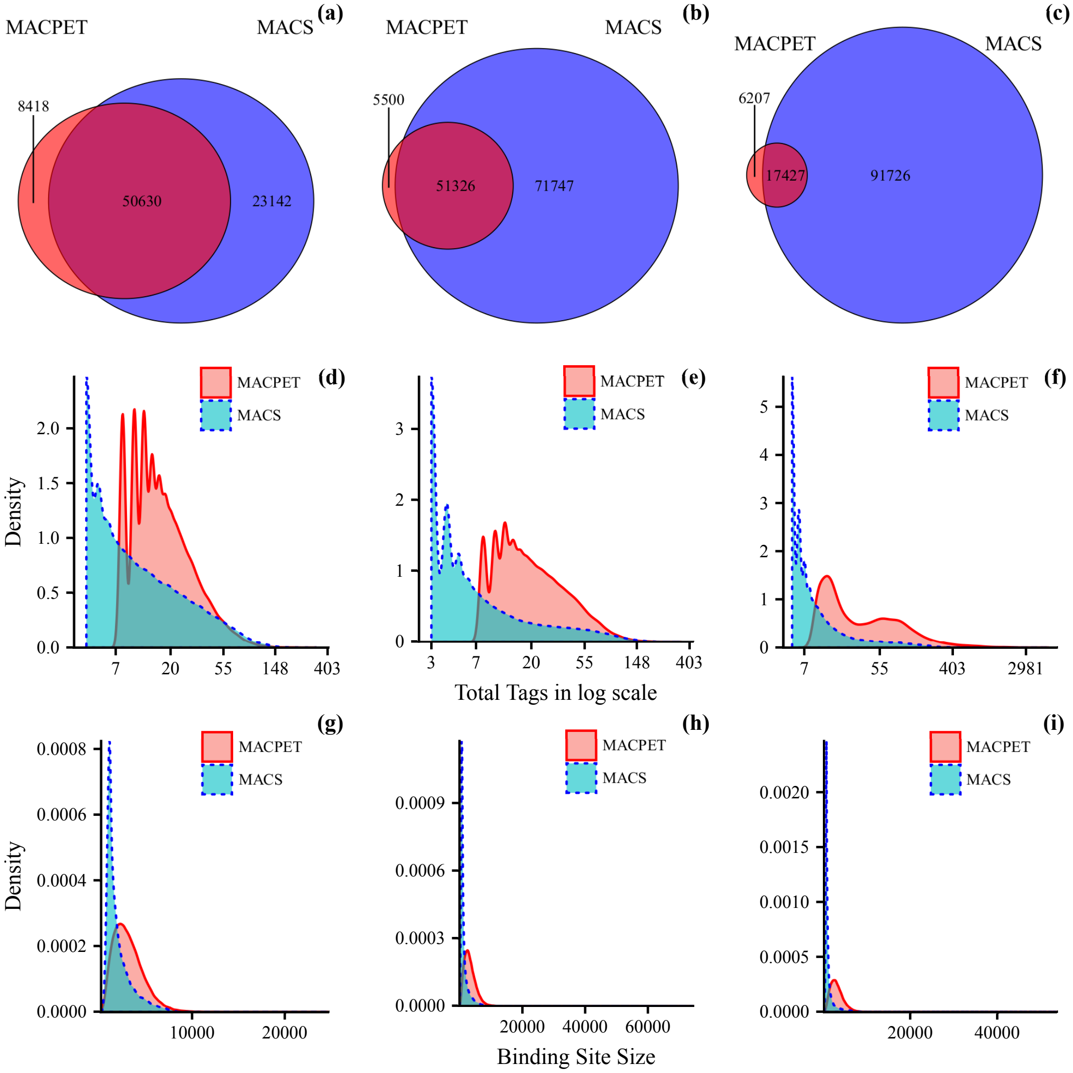

Fig. S7: Comparison of significant binding sites. (a-c) Venn diagrams of the significant PBSs for MACPET and MACS for (a) POL2 (K562), (b) H3K4me1 (K562), (c) H3K27ac (K562). (d-f) densities for the total number of tags in each significant PBSs from MACPET and MACS for (d) POL2 (K562), (e) H3K4me1 (K562), (f) H3K27ac (K562). The x-axis is the total tags in log scale and the y-axis is the density of the total tags. (g-i) densities for the sizes of the significant PBSs from MACPET and MACS for (g) POL2 (K562), (h) H3K4me1 (K562), (i) H3K27ac (K562). The x-axis represents the sizes of the significant PBSs and the y-axis shows their density.

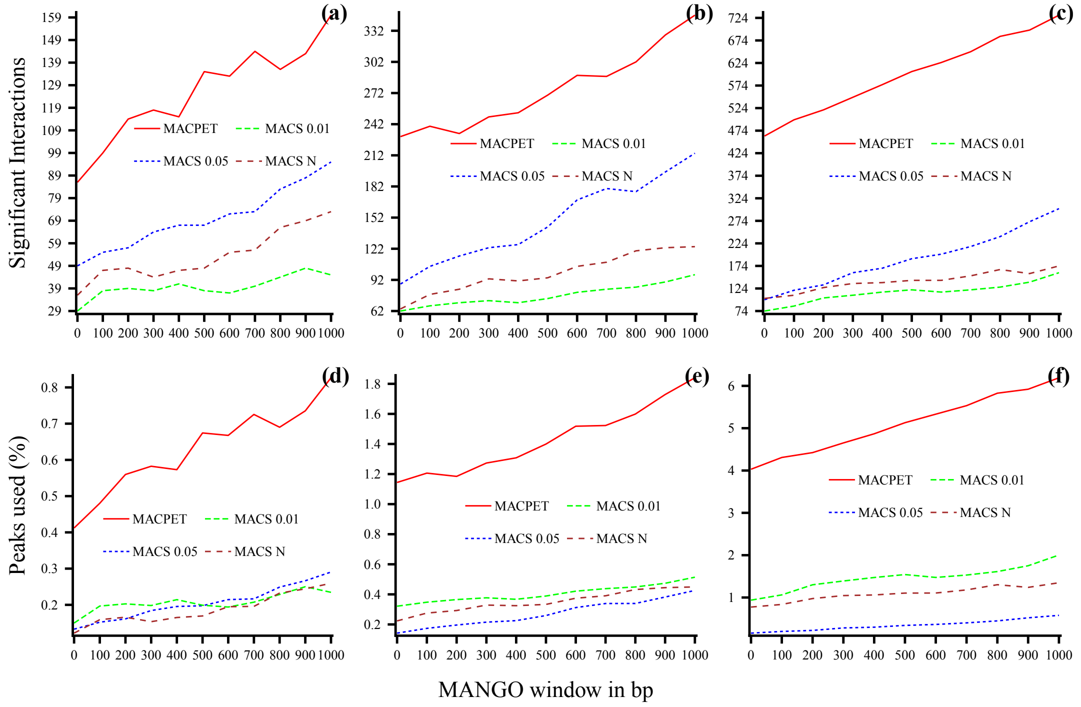

Fig. S8: Comparison for MANGO interactions. Comparison of MANGO interaction results between significant PBSs from MACPET (peaks' FDR$<$ 0.05) and MACS (peaks' FDR$<$ 0.05, FDR$<$ 0.01 and top N most significant peaks), for different PBSs extension windows. For all the plots, the x-axis is the number of bp. Each PBS interval was extended from either side before running MANGO. (a-c) total significant interactions (y-axis) for (a) POL2 (K562), (b) H3K4me1 (K562), (c) H3K27ac (K562). (d-f) proportion of significant PBSs involved in significant interactions (y-axis) for (d) POL2 (K562), (e) H3K4me1 (K562), (f) H3K27ac (K562).

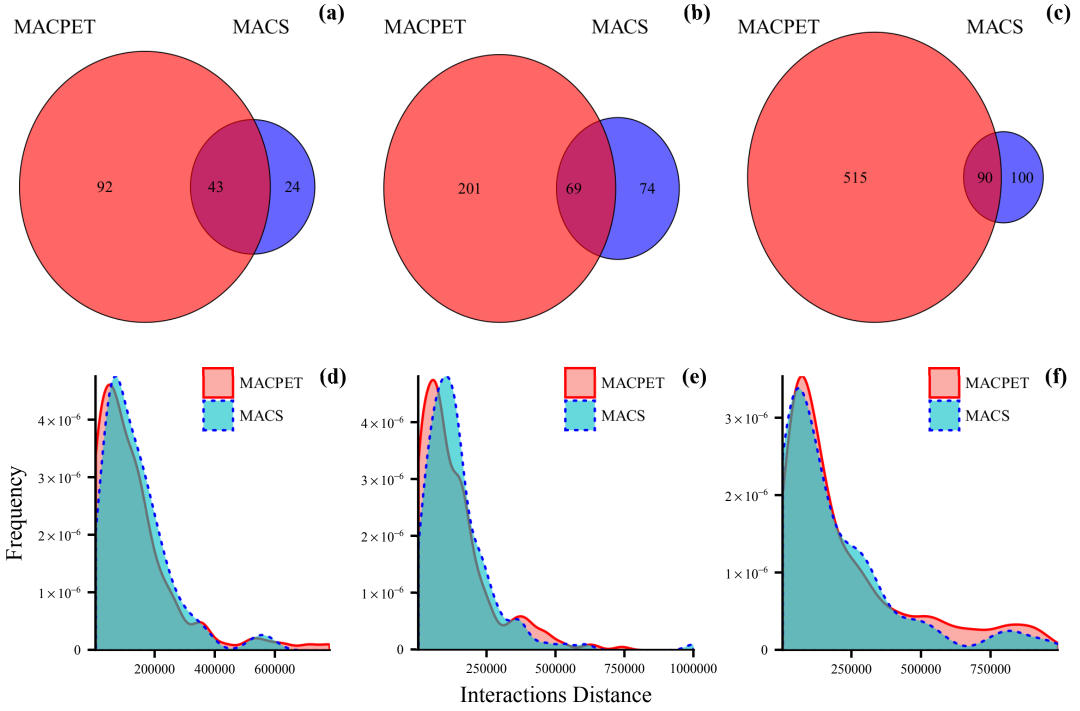

Fig. S9: Comparison for MANGO interactions of 500 bp window extension. (a-c) Venn diagrams from significant interactions for a 500 bp extension window for the significant PBSs from MACPET and MACS for (a) POL2 (K562), (b) H3K4me1 (K562), (c) H3K27ac (K562). (d-f) density plots for the sizes of the significant intra-chromosomal interactions from MACPET and MACS for (d) POL2 (K562), (e) H3K4me1 (K562), (f) H3K27ac (K562). The x-axis represents the distances of the intra-chromosomal interactions and the y-axis shows their density.
